# The CXCL16/CXCR6 axis is linked to immune effector cell-associated neurotoxicity in chimeric antigen receptor (CAR) T cell therapy

**DOI:** 10.1186/s13073-025-01498-6

**Published:** 2025-06-30

**Authors:** I-Na Lu, Louisa Müller-Miny, Carolin Krekeler, Phyllis Fung-Yi Cheung, Georgia Antonopoulou, Astrid Jeibmann, Andreas Schulte-Mecklenbeck, Kornelius Kerl, Simon Call, Christian Reicherts, Annalen Bleckmann, Matthias Stelljes, Georg Lenz, Heinz Wiendl, Gerd Meyer zu Hörste, Oliver M. Grauer

**Affiliations:** 1 Department of Neurology With Institute of Translational Neurology, University Hospital Münster, Albert-Schweitzer-Campus 1, Bldg A1, Münster, 48149 Germany; 2 Department of Pediatric Hematology and Oncology, University Children’s Hospital Münster, Münster, Germany; 3 Department of Medicine A, Hematology, Oncology, and Pneumology, University Hospital Münster, Münster, Germany; 4 Spatiotemporal Tumor Heterogeneity, German Cancer Consortium (DKTK), partner site Essen, a partnership between German Cancer Research Center (DKFZ) and University Hospital Essen, Essen, Germany; 5 Bridge Institute of Experimental Tumor Therapy, West German Cancer Center, University Hospital Essen, University of Duisburg-Essen, Essen, Germany; 6 Division of Solid Tumor Translational Oncology, DKTK, partner site Essen, a partnership between German Cancer Research Center (DKFZ) and University Hospital Essen, Essen, Germany; 7 Institute of Neuropathology, University Hospital Münster, Münster, Germany; 8 Department of Neurology and Neurophysiology, University Hospital Freiburg, Freiburg, Germany

## Abstract

**Background:**

Immune effector cell-associated neurotoxicity syndrome (ICANS) is a common and potentially life-threatening complication of chimeric antigen receptor (CAR) T cell therapy. The underlying mechanisms of ICANS remain incompletely understood and are unlikely to be explained by cytokine excess alone.

**Methods:**

We analyzed paired peripheral blood and cerebrospinal fluid (CSF) samples from CAR T cell–treated patients who developed ICANS (*n* = 11) within 5–21 days post-infusion. ICANS severity was graded as follows: grade 1 (*n* = 3), grade 2 (*n* = 4), grade 3 (*n* = 1), and grade 4 (*n* = 3). Control samples were obtained from patients with idiopathic intracranial hypertension, functional neurological disorders, and multiple sclerosis. We employed single-cell RNA sequencing (scRNA-seq) and flow cytometry to profile immune cell populations and performed multi-modal spatial transcriptomics and immunofluorescence on postmortem choroid plexus and brain tissue from a patient with fatal grade 4 ICANS.

**Results:**

We identified a distinct population of proliferating, cytotoxic T cells characterized by CXCR6 expression, enriched in CD4 + CAR T cells and predominantly localized in ICANS CSF. These CXCR6 + T cells were largely absent from control CSF samples. Spatial mapping of postmortem brain tissue revealed widespread infiltration of myeloid cells and a striking spatial association between CXCR6 + T cells and CXCL16-expressing myeloid cells in both the choroid plexus and brain parenchyma. Notably, CSF levels of CXCL16 positively correlated with ICANS severity across the cohort, from grade 1 to grade 4.

**Conclusions:**

Our findings implicate the CXCL16/CXCR6 axis in the recruitment of cytotoxic CAR CD4 + T cells to the central nervous system (CNS) during ICANS. This interaction may be linked to neuroinflammatory processes and severity stratification in ICANS pathogenesis. These results provide a mechanistic rationale for exploring CXCL16/CXCR6 as a potential biomarker and therapeutic target in CAR T cell-associated neurotoxicity.

**Supplementary Information:**

The online version contains supplementary material available at 10.1186/s13073-025-01498-6.

## Background

CAR T cell therapy can induce multiple adverse effects, among which cytokine release syndrome (CRS) and immune effector cell-associated neurotoxicity syndrome (ICANS) are especially threatening and potentially lethal [1–3]. Anti-CD19 targeting CAR T cell treatment for leukemia and non-Hodgkin’s lymphoma has been associated with severe ICANS, including fatal cerebral edema [4–6]. However, despite its critical significance, ICANS remains poorly understood and current management strategies primarily revolve around corticosteroids [2] and blockade of interleukin-6 receptor (IL-6R) and interleukin-1 receptor (IL-1R), utilizing tocilizumab [7] and anakinra [8]. CAR T cells can migrate and persist in cerebrospinal fluid (CSF) [9], but whether activated CAR T cells in the CSF directly trigger ICANS remains unclear. Some studies do not find any correlation between CAR T cell levels and ICANS severity [10], while others suggest that neurotoxic severity might be more closely related to CSF cytokine levels than to CSF CAR T cell levels [11].

The pathophysiology of ICANS involves multiple contributing factors. Emerging evidence emphasizes the role of activated endothelial and myeloid cells in the development of ICANS, with increased blood–brain barrier (BBB) permeability facilitating the infiltration of peripheral immune cells and cytokines into the CNS [6, 12]. This infiltration may trigger local inflammation, astrocyte injury, and microglial activation. It has been shown that CAR T cells may target cerebral CD19-expressing pericytes, thereby disrupting the BBB and further exacerbating ICANS [13]. However, neurotoxicity is also observed in CAR T-cell therapies targeting B-cell maturation antigen (BCMA) [14], indicating that the syndrome is not solely dependent on the target antigen. We analyzed ICANS patients and, in line with previous findings [6, 10, 12], elevated serum IL-6 levels correlated with CRS (Additional file 1: Fig. S1E), and CRS rapidly resolved after administration of the IL-6 receptor blocker tocilizumab [15]. Nonetheless, while tocilizumab demonstrates efficacy in managing CRS, IL-6 blockade does not consistently prevent ICANS development and its impact on ICANS remains limited in most cases [6, 10, 16]. Given the lack of clarity, we hypothesized that analyzing CSF cells from ICANS patients could provide a deeper insight into the pathophysiology of ICANS. Using scRNA-seq, we generated a transcriptional map of CSF immune cells in patients undergoing CAR T-cell therapy, uncovering a critical role of the CXCL16/CXCR6 axis in the development of ICANS.

## Methods

### Patients

ICANS patients (*n* = 11) were admitted to the University Hospital Muenster between December 2022 and January 2025. All ICANS patients received treatment utilizing commercially available CAR-T cell products (*axi-cel*, *brexu-cel*, *cilta-cel*). Control cohorts include patients with multiple sclerosis (*n* = 8), idiopathic intracranial hypertension (IIH) (*n* = 4), and functional neurological disorder (FND) (*n* = 3) admitted to the University Hospital Münster between November 2011 and June 2024. Patients were diagnosed based on the current diagnostic criteria [17, 18] and in case of FND [19, 20] by excluding other neurological diseases in addition to considering patients’ clinical signs and symptoms. Prior to enrollment, written informed consent was obtained from each participant, following approval from the Ethics Committee of the Board of Physicians of the Region Westfalen-Lippe and the University of Münster (reference: 2010–262-f-S, 2015–522-f-S, 2021–198-f-S, 2024–345-f-S). Clinical data and CSF samples were collected in accordance with ethical guidelines and the principles of the Helsinki Declaration. Cohort details and clinical characteristics are provided in Additional file 2: Table S1. The data are presented in an anonymized form to avoid potentially identifying the individuals involved in this study. Within our clinical cohort (scRNA-Seq, flow cytometry, ELISA, and autopsy material), ICANS patients were initially diagnosed with a diffuse large B cell lymphoma (5/11), a B precursor acute lymphoblastic leukemia (1/11), a highly malignant B cell lymphoma (1/11), or a mantle cell lymphoma (2/11) based on the respective diagnostic criteria. One patient was diagnosed with a multiple myeloma (1/11) and received consecutively anti-BCMA CAR T cell therapy (*cilta-cel*, Carvykti®). Most ICANS patients (7/11) were in Ann Arbor stages of ≥ 3 with a poor prognosis using the international prognostic index (IPI) [21, 22]. All other ICANS patients received treatment with *brexu-cel* (Tecartus®) or *axi-cel* (Yescarta®) after lymphodepletion with cyclophosphamide/fludarabine. After CAR infusion (day 0), patients were monitored with a CAR-specific neurological symptom checklist (NSC) and treated according to clinical standard operation procedures and current guidelines [23–25].

### Sample preparation

All lumbar punctures were conducted under strict sterile conditions and performed for clinical reasons. CSF samples were meticulously collected in sterile tubes utilizing the Sprotte spinal needle, with processing within a 2-h window post lumbar puncture to uphold sample integrity. Upon collection, CSF was carefully transferred into round-bottom polypropylene tubes and subsequently centrifuged at 300 × g for 10 min. The resulting cell-free supernatants were delicately withdrawn using a pipette, then preserved at − 80 °C for subsequent proteomic analysis. The CSF-cell pellet was resuspended in autologous CSF and gently diluted into 500 μL of RPMI-1640 supplemented with 40% FBS. For cryopreservation, the CSF cell suspension was slowly diluted dropwise with 500 μL of pre-chilled freezing medium comprising RPMI-1640 supplemented with 40% FBS and 20% DMSO, as previously shown [26, 27]. Subsequently, the CSF sample was expeditiously placed within a freezing container (Mister Frosty, Thermo Fisher) and stored overnight in a − 80 °C freezer prior to transfer to liquid nitrogen (LN) storage. For the thawing of cryopreserved CSF cells, the samples were carefully retrieved from the LN and thawed at 37 °C for 1–2 min, allowing for the presence of a tiny ice crystal within the cryotube. The cells were then diluted 1:10 in PBS supplemented with 10% FBS and subjected to centrifugation at 300 × g for 10 min at 4 °C. After discarding the supernatant, the cells were resuspended in 50 μL of PBS, with 10 μL reserved for cell counting purposes. The remaining cells were then processed for 5′-single cell RNA sequencing.

### Single-cell sequencing and data preprocessing

Single-cell suspensions were loaded onto the Chromium Single Cell Controller according to the manufacturer’s instructions. The scRNA-seq libraries were processed using Chromium Next GEM Single Cell 5′ Reagent Kits v2 (10× Genomics) and coupled scTCR-seq libraries were successfully obtained from all samples except one using Chromium single-cell V(D)J enrichment kit (human T cell) (10× Genomics). Quantification was conducted with precision using the TapeStation High Sensitivity D1000 kit (Agilent) to ascertain the average fragment size and library concentration. Subsequently, the libraries underwent normalization, dilution, and were sequenced on a local Nextseq 2000 using the P3 100 cycle kit with a 28–10-10–90 read setup. To process the sequencing data, a customized reference was constructed by incorporating the previously characterized CAR gene sequences [28] for both *axi-cel* and *brexu-cel*, which originated from msgv-fmc63-28z [29], into the GRCh38 reference genome. This custom reference was prepared using Cellranger mkref (version 7.2.0) adhered to the manufacturer’s instructions. Raw bcl files were then demultiplexed using the cellranger mkfastq pipeline. Subsequent read alignments and transcript counting were done individually for each sample using the cellranger count pipeline using the custom reference described above.

### Single-cell analysis

Single-cell analysis was conducted using Seurat v5 [30] in R version 4.3.2. Pooled samples from individuals with IIH were demultiplexed employing Vireo [31], a genotype-based demultiplexing method. The cellranger matrix was loaded into Seurat. SoupX [32] 1.6.2 was carried out with the automatic method using clustering information to reduce ambient RNA expression. Cells were filtered for less than 5% mitochondrial RNA transcripts, a minimum of 200 genes and a maximum of 3500 feature genes for each sample based on inspection of the QC plots (Additional file 2: Table S2). Next, we normalized the samples separately with SCTransform v2 [33] and then removed the batch effect with Harmony [34]. Clusters and UMAP embeddings were computed based on the Harmony reduction within Seurat using a resolution of 1.2 and 40 dimensions. The resulting 23 clusters were subsequently categorized into 6 distinct cell types based on their respective marker genes. Predicted classification of each cell in either G2M, S, or G1 cell-cycle phase was done by CellCycleScoring function, and fractions of CAR_transcript- and CXCR6-expressing cells were calculated through the PercentageFeatureSet function within the Seurat package. Dot plots and feature plots were generated with Seurat functions based on the log-normalized data. CellChat [35] R package was utilized to investigate and visualize cell–cell communication and ligand-receptor interactions among different CSF cells using standard package functions.

### Analysis of differential cell abundances and gene expression

Differential abundance (DA) was determined with the propeller [36] tool, which is part of speckle v1.0.0. Briefly, we transformed the cell type proportions with a logit function. When comparing ICANS_onset to IIH, we build a linear model adjusting for gender and age. We computed significance based on the robust empirical Bayes method from limma. *p* values were adjusted with the Benjamini–Hochberg method. Following this, differentially expressed genes (DEGs) analysis at the pseudobulk level was conducted between ICANS_onset and IIH as well as between CXCR6 + and CXCR6 − cells employing DESeq2 through the FindMarkers function within the Seurat package. The DA and DEGs results were visualized with ggplot2.

### Gene set enrichment analysis (GSEA)

Significantly regulated genes were identified by adjusted *p* value < 0.05 following the DEGs analysis and were further input into GSEApy [37] version 1.1.2 in Python version 3.10.0 for enrichment analysis using two-pathway gene sets, including MSigDB_Hallmark_2020 and KEGG_2021_Human. The enrichment scores across all individual cells and groups were computed by the enrichIt function of the escape package and were visualized with dittoSeq package.

### Single-cell immune repertoire analysis

Single-cell T cell receptor sequencing (scTCR) data was analyzed using scRepertoire v2.0 [38] and immunarch v0.9.1 [39]. The filtered outputs of CellRanger vdj were imported, and the TCR heavy and light chains were combined in each cell based on their barcodes. Cells with more than two immune receptor chains were removed from the dataset. Clonotypes were called using the CDR3 amino acid sequence.

### Flow cytometry

We screened all patients who were admitted to the University Hospital Münster between 2022 and 2025 and received CAR T cell therapy. All samples collected during regular working hours at the center in Münster were promptly analyzed by flow cytometry using a Navios flow cytometer (Beckman Coulter). Briefly, blood cells were lysed using VersaLyse buffer and blood and CSF cells were stained using the following anti-human antibodies (clone names indicated): CD3 (SK7); NCAM (HCD56), CD69 (FN50), Granzyme A (CB9), CXCR6 (K041E5); Beckman Coulter: CD4 (SFCI12T4D11), CD8 (A99019), CD45RA (A07786), CD45 (B36294); Miltenyi Biotec CD19-CAR FMC63 Idiotype Antibody (REA1297). The gating scheme is depicted in Additional file 1: Fig. S8A and B. Monocytes were analyzed by spectral cytometry using a Aurora flow cytometer (Cytek). Blood and CSF cells were stained using the following anti-human antibodies (clone names indicated): BD OptiBuild: CD14 (63D3.rMAb); Beckman Coulter CD16 (B49216), Miltenyi Biotec: CD40 (REA733), Sony: CD49d (9F10). The gating scheme is depicted in Additional file 1: Fig. S11I. Cell population size was defined as the number of gated cell events relative to the events of the corresponding parent gate.

### ELISA

An enzyme-linked immunosorbent assay (ELISA) (R&D Systems Human CXCL16 ELISA Kit-Quantikine #DCX160) was performed according to the manufacturer’s instructions. In brief, CSF supernatants were processed as shown previously here and aliquots were stored at − 80 °C. For analysis, samples were thawed and after a dilution series used in a final dilution of 1:4 in a 96-well plate. Samples were measured in technical duplicates in a Tecan Plate reader Infinite M200pro (Tecan) at a wavelength of 450 nm with a reference at 620 nm. The aliquots used have not been repeatedly frozen/thawed prior to this study. The intra-assay variance was at 2.6%.

### Immunohistochemistry

Seven-color multiplexed immunohistochemistry was performed on one postmortem formalin-fixed paraffin-embedded (FFPE) ICANS brain sample with lethal ICANS grade 4 syndrome using the Opal multiplex system [40] (Akoya Biosciences). In brief, FFPE sections of 2-μm thickness were deparaffinized and then fixed with 4% paraformaldehyde prior to antigen retrieval by heat-induced epitope retrieval using citrate buffer (pH6) or Tris/EDTA (pH9). After cooling down from antigen retrieval, slides were immersed in alkaline hydrogen peroxide solution (pH11.5) in transparent plasticware and exposed to white light by sandwiching the immersed slides between two light-emitting diode (LED) panels. The slides were incubated for 45 min, after that the solution was discarded and replaced by freshly prepared hydrogen peroxide solution for another 45 min LED exposure. Slides were then washed with PBS and ready for subsequent staining. Each section was put through several sequential rounds of staining (Additional file 2: Table S3); each included endogenous peroxidase blocking and non-specific protein blocking, followed by primary Ab and corresponding secondary horseradish peroxidase-conjugated polymer (Zytomed Systems, Germany or Akoya Biosciences). Each horseradish peroxidase-conjugated polymer mediated the covalent binding of different fluorophores using tyramide signal amplification. Such covalent reaction was followed by additional antigen retrieval in heated citric buffer (pH6) or Tris/EDTA (pH9) for 10 min to remove antibodies before the next round of staining. After all sequential staining reactions, sections were counterstained with DAPI (Vector lab). Slides were subsequently scanned and digitalized by Zeiss AxioScanner Z.1 (Carl Zeiss AG) with × 10 objective magnification. Quantification of co-expressing markers and proximity analysis were performed with HALO (Indica Labs). Four regions of interest (ROIs), each measuring 1000 µm × 1000 µm, were selected within brain parenchyma areas exhibiting T cell infiltration (Additional file 1: Fig. S12A). The proportion of CXCR6 + CD3 + DAPI + cells located within a 100 µm proximity to either CXCL16+ CD11b+ DAPI+ cells or CXCL16− CD11b+ DAPI+ cells was calculated. Cell counts were quantified within each ROI to determine these ratios.

### Spatial gene expression assay and data preprocessing

Five-micrometer FFPE serial sections were placed on standard glass slides and H&E-stained and processed following the manufacturer’s instructions (Visium HD by 10X Genomics). The CytAssist instrument was used to facilitate the transfer of transcriptomic probes from the standard glass slide to the Visium HD Spatial Gene Expression slide with 6.5 × 6.5 mm capture area. Sequencing was performed on the NextSeq 2000 with a 43–10-10–50 read setup. Flow cells were demultiplexed using the mkfastq command in Space Ranger v3.0.1 (10X Genomics), and FASTQs were aligned to the human (GRCh38) reference. Feature plots were generated with Seurat SpatialFeaturePlot function and with ggplot2 using the dataset with 8-µm bins.

## Results

### Clinical characteristics of ICANS patients

Within our study cohort, we included ICANS patients with primary diagnosis of diffuse large B cell lymphoma (5/11), B precursor acute lymphoblastic leukemia (1/11), highly malignant B cell lymphoma (1/11), mantle cell lymphoma (2/11), follicular lymphoma (1/11), and multiple myeloma (1/11) based on the respective diagnostic criteria. CAR treatment was determined based on ASCO guidelines [23] and patients received brexu-cel (3/11), axi-cel (7/11), cilta-cel (1/11) after standardized lymphodepletion (see Additional file 2: Table S1). After CAR infusion (day 0), all patients were monitored using a CAR-specific neurologic symptom checklist (NSC). Patients developed cytokine release syndrome (grade 1 to 3) within 3 days (range 1–5 days) of CAR infusion with signs and symptoms including fever (100%) and hypotension (33%) (Additional file 1: Fig. S1A). Patients in our cohort developed ICANS [23] within 7 days (range 5–21 days) of CAR infusion with varying degrees of severity (ICANS grade 1 (3/11), grade 2 (4/11), grade 3 (1/11), grade 4 (3/11)) (Additional file 2: Table S1). A lumbar puncture was performed for clinical reasons if ICANS was suspected and ICANS patients showed an increased CSF cell count and total protein (Additional file 1: Fig. S1B, C) as described previously [3]. Serum IL-6 levels correlated closely with CRS (Additional file 1: Fig. S1D) as described previously [3].

### Transcriptomic profiling of CSF cells reveals CXCR6 upregulation and proliferative signatures during ICANS

We analyzed CSF samples from five patients (pt. 1–5) at the onset of ICANS (ICANS_onset), including one patient (pt. 1) with longitudinal samples collected before CAR T-cell infusion (pre-CART) and after ICANS remission (ICANS_remission). As a control, we included existing CSF cell data from donors with non-inflammatory idiopathic intracranial hypertension (IIH) (Additional file 2: Table S1).

**Fig. 1:**
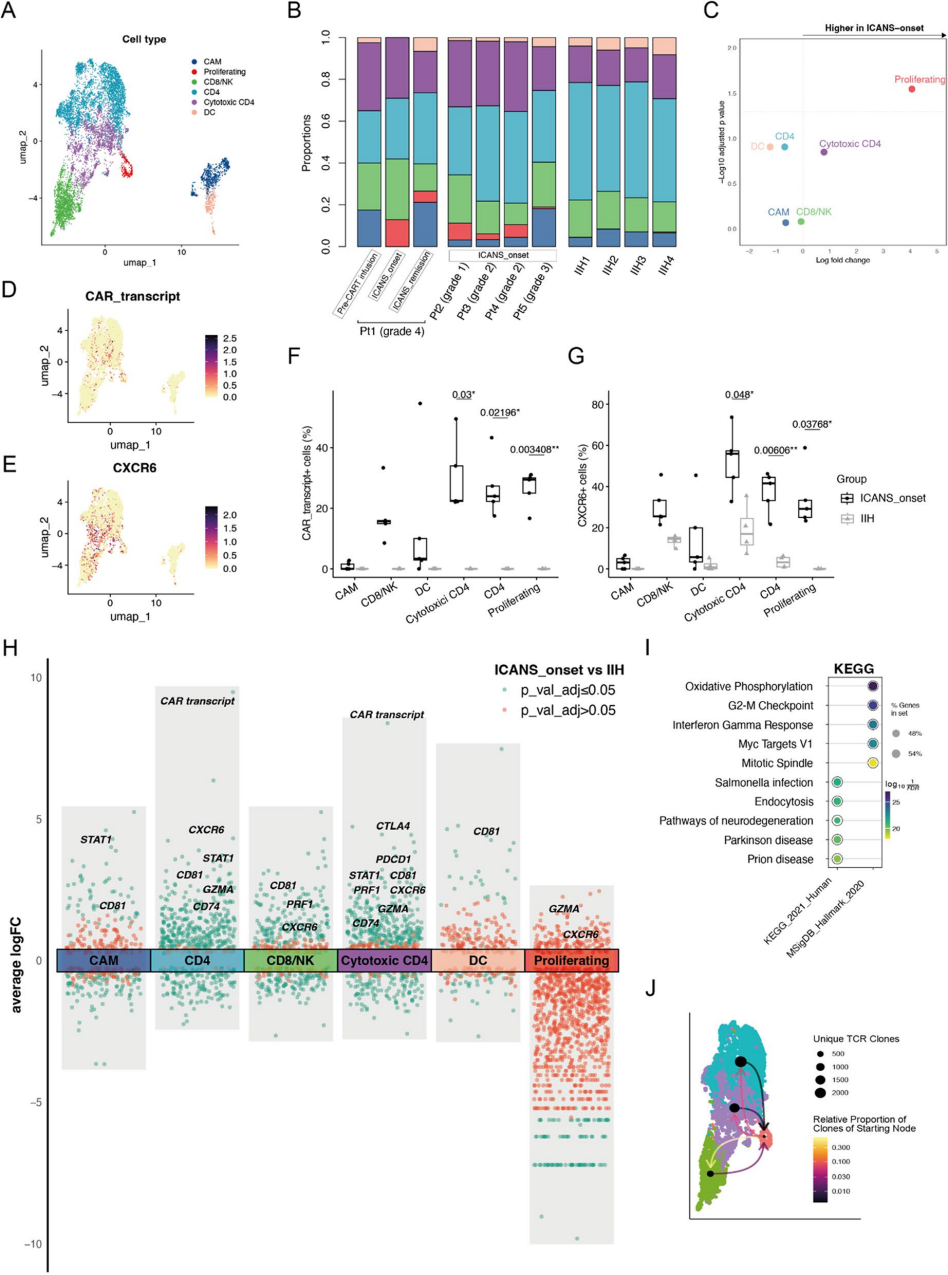
The landscape of immune populations in the CSF of ICANS patients. **A** UMAP plot showing 6 color-coded cell types of CSF cells from ICANS patients (*N* = 7, cell count = 5148) and IIH control donors (*N* = 4, cell count = 3513). DC, dendritic cells; CAM, CNS-associated myeloid cells. **B** Proportions of cells split by samples (*N* = 11). **C** Volcano plot depicting changes in cell type abundances in ICANS_onset (*N* = 5) versus IIH (*N* = 4). A minimum threshold of 25% abundance and a log2 fold change (logFC) of 0.25 were applied. Statistical significance calculated via propeller *t*-test, which uses a moderated *t*-statistic based on the robust empirical Bayes method from limma. *p* values were adjusted with the Benjamini-Hoch method. Logarithmic fold change of cluster abundance is plotted against negative logarithmic adjusted *p* value. **D** Expression of CAR_transcript gene overlaid onto the UMAP. **E** Expression of CXCR6 gene overlaid onto the UMAP. Fraction of CAR_transcript- (**F**) and CXCR6- (**G**) expressing cells per sample stratified by cell types. Each circle/triangle represents a sample. Data are depicted as median, and the lower quartile and upper quartile. Whisker include 1.5 times the interquartile range. Unpaired two samples *t*-test and the Bonferroni method is used to calculate the *p* values. **H** Differential gene expression analysis showing up- and downregulated genes across all six cell types between ICANS_onset (*N* = 5) versus IIH (*N* = 4) samples. *p* values were adjusted with the Benjamini-Hochberg method. **I** Enrichment analysis of differential KEGG pathways in the proliferating, cytotoxic CD4, and CD4 cell types of ICANS_onset (*N* = 5) versus IIH (*N* = 4) samples. **J** Network based on proportions of TCR clones shared between proliferating cells with other T cells. *p* values were adjusted with the Bonferroni method

Clustering the cells allowed for the identification of expected immune cell types based on canonical markers (Fig. 1A and Additional file 1: Fig. S2A, B). CSF cells from ICANS patients interspersed with those from IIH controls (Additional file 1: Fig. S2C). Most clusters, particularly the non-proliferating cell clusters, were derived from both ICANS and IIH donors, suggesting that variability among samples was less pronounced than variability among cell types (Additional file 1: Fig. S2C). We categorized CD4 T cell clusters into two groups based on the percentage of cells expressing the GZMA gene as a proxy of cytotoxicity and activation: cytotoxic CD4 (≥ 70%) and CD4 (< 70%) (Additional file 1: Fig. S2D). After this subsetting, proliferating cells were significantly increased in ICANS_onset samples compared to IIH controls (Fig. 1B, C). While not statistically significant, CSF cells from ICANS_onset patients exhibited a higher proportion of cells in the G2M phase of the cell cycle (Additional file 1: Fig. S3A) and showed enrichment in hallmark gene sets associated with cell proliferation, including G2M checkpoint and E2F targets (Additional file 1: Fig. S3B). These proliferative cells were primarily derived from ICANS_onset samples and predominantly consisted of CD4 T cells (Additional file 1: Fig. S3C). Moreover, cells with an in silico detected CAR transcript (Methods) were mostly present in T cell clusters within ICANS_onset samples (Fig. 1D, F). As expected, no CAR transcripts were detected in IIH samples (Fig. 1F).

In addition, we conducted differentially expressed genes (DEGs) analysis between ICANS_onset and IIH samples for each annotated cell type to identify transcriptional alterations in ICANS. Consistently, CXCR6 was upregulated in ICANS patients compared to IIH controls, both in terms of gene expression levels (Fig. 1E, Additional file 1: Fig. S4A) and the percentage of cells expressing CXCR6 was higher in ICANS patients across multiple T cell subsets, including proliferating, cytotoxic CD4, and CD4 cells (Fig. 1G, H). Additionally, we found a significant positive correlation between CXCR6 gene expression and the granzyme gene, GZMA, across all T cell subsets during the onset of ICANS, suggesting that upregulation of CXCR6 is associated with increased T cell cytotoxicity (Additional file 1: Fig. S4B). KEGG pathway enrichment analysis revealed several top enriched pathways implicated in various aspects of ICANS pathogenesis. Notably, pathways associated with increased energy demand (oxidative phosphorylation), cell-cycle regulation (G2M checkpoint, Myc target V1, mitotic spindle), and inflammatory responses (interferon gamma response, endocytosis) were among the top enriched pathways identified in the ICANS_onset CSF samples (Fig. 1I). These pathways are known to be associated with T cell cytotoxic responses, suggesting their potential involvement in ICANS.

We next reconstructed single-cell T cell receptor (TCR) sequence information from these CSF samples. Our analysis revealed a modest predominance of larger clones (10% > clone frequency > 1%) within the proliferating and CD8 T cell clusters in ICANS_onset samples (Additional file 1: Fig. S5A–E). Further analysis of proliferating cells from ICANS_onset patients (with nearly no proliferating cells observed in IIH controls) revealed that CAR + proliferating cells contained a higher proportion of large clonal expansions compared to their CAR − counterparts (Additional file 1: Fig. S5F). Clonal network analysis indicated that proliferating cells shared a higher clonal proportion with CD8 T cells than with the other two CD4 T cell clusters (Fig. 1J). This phenomenon of CD4 T cell proliferation without significant clonal expansion suggests that the observed increase in CD4 + proliferating cells is driven not by the rapid replication of a few specific clones but by active division across a diverse array of CD4 T cell clones. Moreover, comparative analysis of clonal diversity across samples and groups revealed significantly higher TCR diversity, as measured by the Chao1 index, in ICANS_onset patients (Additional file 1: Fig. S5G, H), corroborating our observations of diverse clonal contributions to the expanded CD4 T cell population.

### Intercellular communication and molecular pathways in cytotoxic CD4 T cells during ICANS

**Fig. 2:**
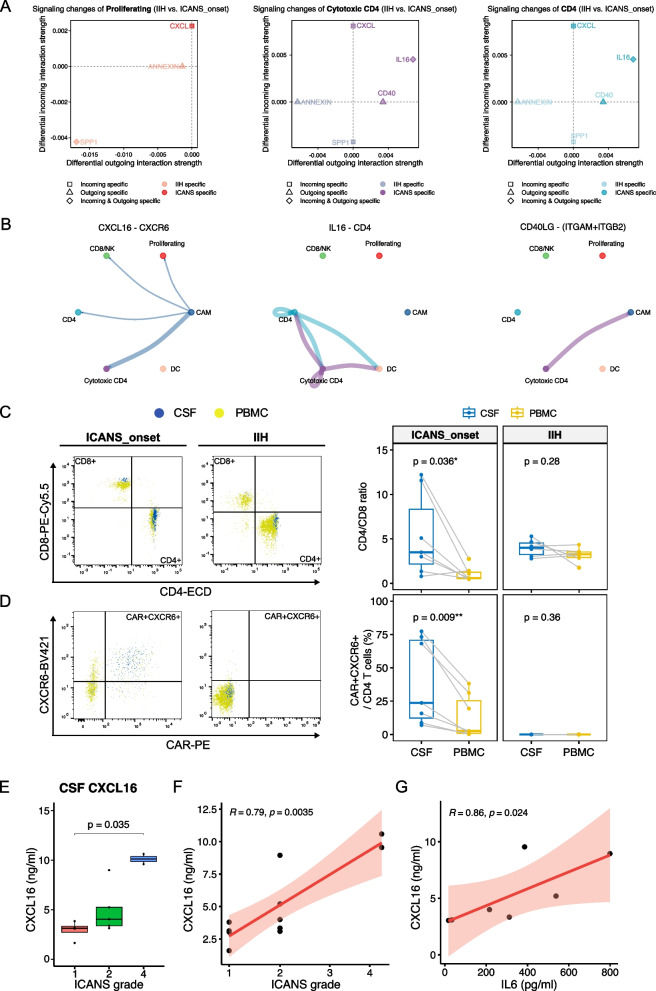
CXCL16-CXCR6 axis mediates T cell infiltration into the CNS during ICANS. **A** Altered proliferating-associated, cytotoxic CD4-associated, and CD4-associated signaling pathways in IIH versus ICANS_onset. The x and y axes indicate differential outgoing and incoming interaction strengths, respectively. **B** Circle plots of the significant interactions (ligand-receptor pairs) in the selected ICANS specific signaling networks from **A**, including CXCL16-CXCR6 of the CXCL signaling pathway, IL16-CD4 of the IL16 signaling pathway, and CD40LG-(ITGAM + ITGB2) of the CD40 signaling pathway. **C** Representative image and comparison of CD4/CD8 ratios between paired CSF and PBMC between paired CSF and PBMC by flow cytometry. **D** The percentage of CAR + CXCR6 + cells within CD4 T cells between paired CSF and PBMC, segmented by patient groups: ICANS_onset (*N* = 7) and IIH control (*N* = 7). Representative Images depicted as a bivariate overlay plot of CSF and PBMC samples of ICANS_onset and IIH. Each circle represents a sample. Data are depicted as median, and the lower quartile and upper quartile. Whiskers include 1.5 times the interquartile range. Statistical significance calculated via paired *t*-test with the Holm-Bonferroni correction method for *p* value adjustments. **E** Boxplot representing CXCL16 concentration (ng/ml) in the CSF in ICANS patient samples (*N* = 10) across different ICANS grades. Data are depicted as median with lower and upper quartiles. Whiskers include 1.5 times the interquartile range. Statistical significance is calculated with a Kruskal–Wallis test and post hoc Dunn’s test for *p* value adjustment. **F** Scatter plots depicting spearman correlations of CSF CXCL16 level with clinical ICANS severity in ICANS_onset patients (*N* = 10) and **G** CSF IL-6 levels, if available (*N* = 7) with ICANS grade

We further explored the intercellular communication within the cell clusters. Differential communication analysis revealed three principal signaling alterations in cytotoxic CD4 T cells, specifically involving the CXCL, IL16, and CD40 pathways (Fig. 2A). These changes were predominantly driven by the CXCL16-CXCR6, IL16-CD4, and CD40LG-(ITGAM + ITGB2) axes, respectively (Fig. 2B). Notably, the top differential incoming signaling was mediated by CXCL for proliferating, cytotoxic CD4, and CD4 cells in ICANS_onset CSF (Fig. 2A), and the potential source of this CXCL signal (CXCL16) was identified to be CNS-associated myeloid cells (CAM) (Fig. 2B). Indeed, when we examined the temporal dynamics of the expression of CXCL16 chemokine as well as fractions of CAR_transcript + and CXCR6 + cells from pre-CART infusion to ICANS_onset and ICANS_remission among the proliferating, cytotoxic CD4, and CD4 cells of one patient (pt. 1, Additional file 2: Table S1), we observed peaking expression around the onset of ICANS over time (Additional file 1: Fig. S6A, B). The expression levels of GZMA, GZMB, and CXCR6 genes decreased in ICANS_remission compared to ICANS_onset (Additional file 1: Fig. S6C). The temporal dynamics of CXCR6-expressing cytotoxic T cells thus mirror the development of ICANS suggesting their mechanistic relevance in the disease.

To investigate ICANS-associated alterations across different compartments and confirm transcriptional data on protein level, we next performed multicolor flow cytometry on paired CSF and blood samples from seven ICANS patients at disease onset (Additional file 2: Table S1). Consistent with our scRNA-seq findings, we observed a significantly higher proportion of CD4 T cells in the CSF compared to the blood of ICANS patients, reflected by increased CD4/CD8 ratios in the CSF (Fig. 2C, Additional file 1: Fig. S7A, B). This enrichment of CD4 T cells within the CNS compartment was not present in most IIH donors without neuroinflammation, but evident in the CAR T cell fraction (Additional file 1: Fig. S7C). Moreover, a significantly higher percentage of specifically CAR + CXCR6 + CD4 T cells was detected in the CSF of ICANS patients compared to their paired blood samples, while CAR + CXCR6 + CD8 T cells did not show a difference (Fig. 2D, Additional file 1: Figs. S7D–F, S8A, B). Longitudinal analysis of T cells from one patient (pt. 8, Additional file 2: Table S1) revealed the appearance of CAR + CXCR6 + CD4 and CD8 blood T cells after passing the CRS maximum with a selective increase of CAR + CXCR6 + CD4 T cells in the CSF during ICANS (Additional file 1: Fig. S8B, C), highlighting the critical role of CXCR6 as a specific receptor that drives the preferential accumulation of cytotoxic CD4 CAR T cells in the CNS during ICANS onset. Indeed, although CD4 CAR T cells in the CSF presented a more activated phenotype by expressing other activation markers such as CD69 and GZMA compared to the blood (Additional file 1: Fig. S9A–D), only CXCR6 showed a specific increase on CAR+ T cells within the CSF compared to CAR − T cells (Additional file 1: Fig. S9E–G).

CXCR6 is activated by its ligand CXCL16. We detected a significant correlation between CSF CXCL16 levels and ICANS severity (Fig. 2E, F), with no similar association for CSF IL-6 levels (Additional file 1: Fig. S10), despite a correlation between IL-6 and CXCL16 levels (Fig. 2G). These results suggest a divergence between systemic and CNS inflammation, implicating the CXCL16/CXCR6 axis as a specific contributor to ICANS pathophysiology and soluble CXCL16 as a possible versatile future disease biomarker.

### Elucidating the role of the CXCL16-CXCR6 axis in T cell infiltration and ICANS pathology

**Fig. 3:**
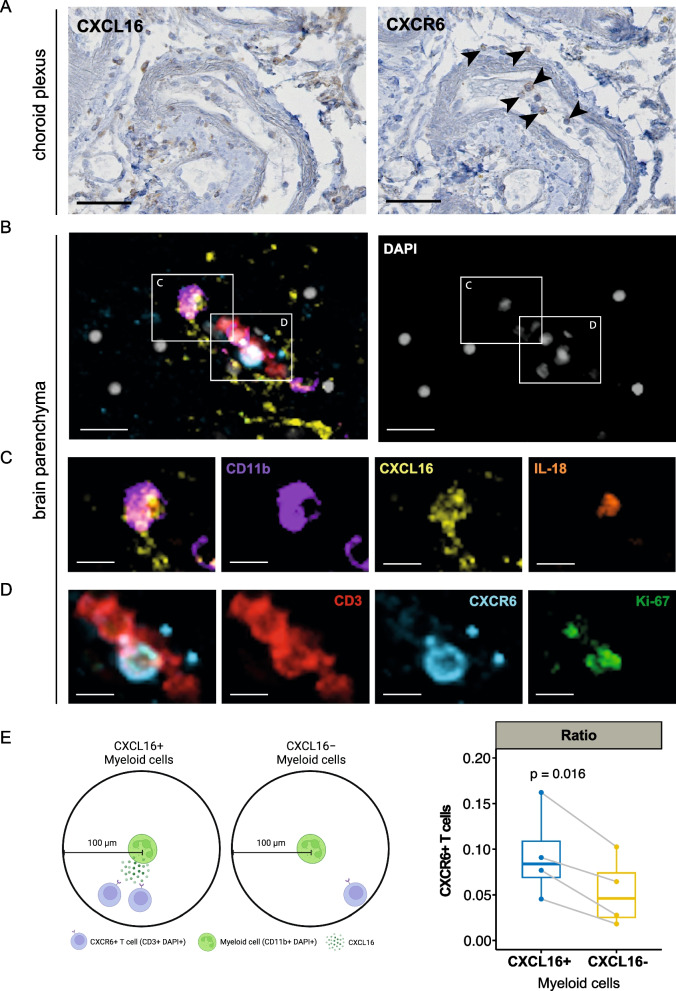
CXCR6 + T cells and CXCL16 + myeloid cells in the choroid plexus and brain parenchyma of one postmortem ICANS patient. **A** CXCL16 and CXCR6 immunohistochemistry of the ICANS choroid plexus (scale bar, 50 µm). **B** Multiplexed immunofluorescence staining (left) and DAPI single staining of one representative postmortem ICANS brain parenchyma. White reticular boxes indicate the areas detailed in **C** and **D**. CD3 (red), CXCR6 (light blue), DAPI (white), CXCL16 (yellow), and CD11b (violet). Scale bar: 20 μm. **C** High-resolution image highlighting CD11b + CXCL16 + cells in **B**, stained for CD11b (violet), CXCL16 (yellow), and IL-18 (orange). Scale bar: 10 μm. **D** High-resolution image highlighting CD3 + CXCR6 + cells in **B**, stained for CD3 (red), CXCR6 (light blue), and Ki-67 (green). Scale bar: 10 μm. **E** Ratio of CXCR6 + T cells to CXCL16 + or CXCL16 − myeloid cells within a 100 μm radial distance in four brain parenchyma regions (1000 × 1000 μm areas) from a postmortem ICANS brain (*N* = 1). Data are depicted as median, and the lower quartile and upper quartile. Whisker include 1.5 times the interquartile range. Statistical significance calculated via paired *t*-test with the Holm-Bonferroni correction method for *p* value adjustments. The diagram was created in BioRender. Lu, I. (2025) https://BioRender.com/w42c592

We aimed to confirm the presence of CXCL16 ligand and CXCR6+ T cells at the protein level in ICANS-affected brain tissues, and conducted conventional immunohistochemistry on choroid plexus samples from an ICANS patient who had died following severe cerebral edema (pt. 11, Additional file 2: Table S1). This demonstrated the presence of both CXCR6 + and CXCL16 + cells (Fig. 3A). Further investigation into the cellular sources of the CXCL16 signal was carried out using high-resolution spatial transcriptomic analysis (Methods), which highlighted extensive CXCL16 expression across the choroid plexus (Additional file 1: Fig. S11A). This CXCL16 expression was spatially associated with myeloid cell markers such as CD14 (Additional file 1: Fig. S11B), along with activation markers CD40 (Additional file 1: Fig. S11C) and CD68 (Additional file 1: Fig. S11D). A substantial fraction of these cells expressed integrins ITGAL (CD11a, Additional file 1: Fig. S11E) and ITGA4 (CD49d, Additional file 1: Fig. S11F), key facilitators of myeloid cell migration across the blood–brain barrier in response to inflammation [41–43], while a lower proportion of cells displayed markers of resident microglia, including CX3CR1 (Additional file 1: Fig. S11G) and TMEM119 (Additional file 1: Fig. S11H). Explorative flow cytometric analysis of an additional ICANS patient (ICANS grade 2, day 5 after brexu-cel therapy) confirmed a cross compartment shift of activated classical monocytes upregulating CD40 and CD49d from blood to the CSF (Additional file 1: Fig. S11I). This suggests that infiltrating myeloid cells, rather than resident microglia, are most likely to produce CXCL16 in the ICANS-affected choroid plexus and thus may attract CXCR6-expressing T cells.

Due to the significant autofluorescence of ependymal cells complicating multiplex immunofluorescence (mIF) staining in choroid plexus tissues, we conducted mIF analysis on postmortem periventricular brain tissues (basal ganglia) harvested from the same ICANS patient (Additional file 1: Fig. S12A). Our results revealed CXCR6 + CD3 + T cells within regions of the brain parenchyma that also exhibited considerable CXCL16 + signals (Fig. 3B–E, Additional file 1: Fig. S12B). Positioned near these T cells were CXCL16 + CD11b + myeloid cells, which also expressed IL-18 (Fig. 3C), a cytokine known to enhance cell-mediated cytotoxicity in neurodegenerative conditions [40]. Additionally, these CXCR6 + CD3 + T cells demonstrated elevated Ki-67 levels, indicative of an active proliferative state congruent with our scRNA-seq data (Fig. 3D). Notably, we observed an elevated proximity ratio of CXCR6 + CD3 + T cells to CXCL16 + CD11b + myeloid cells when compared to CXCL16-CD11b + myeloid cells (Fig. 3E). In addition, by comparing CAR + and CAR − T cells, we verified that both CXCR6 protein and gene expression was significantly increased in CSF CAR-expressing CD4 T cells in ICANS_onset patients (Additional file 1: Figs. S9E, S13). Together, these findings underscore the potential pivotal role of the CXCL16-CXCR6 axis in propagating T cell infiltration into the CNS during ICANS.

## Discussion

Our study provides insights into the cellular and molecular profiles of immune cells within the CSF of ICANS patients undergoing CAR T cell therapy, enhancing our understanding of ICANS pathophysiology and pointing towards potential therapeutic avenues. We noted an enrichment of CD4 CAR T cells in the CSF of ICANS patients, particularly those expressing CXCR6. These cells exhibited a more activated, cytotoxic profile compared to their counterparts in the blood. Our observations suggest that CXCR6 + CAR CD4 T cells may play a role in mediating cytotoxic responses within the CNS, potentially contributing to ICANS by their proximity to CXCL16 + IL-18 + myeloid cells and their apparent proliferative activity. This aligns with the hypothesis that ICANS involves a multifaceted interaction among immune effector cells, cytokine signaling, and the BBB [2].

The association we observed between CXCR6 expression and ICANS severity, together with the temporal dynamics of CXCR6+ CAR T cells during disease onset, hints at the possible significance of the CXCL16/CXCR6 axis in driving neuroinflammation in ICANS. Our findings support existing literature suggesting that systemic cytokine levels, such as IL-6, do not completely explain the severity and progression of neurotoxicity [6, 10, 16]; rather, local inflammatory processes within the CNS, particularly involving the CXCL16/CXCR6 axis, appear to have a more defining role. The identification of CXCR6-expressing cytotoxic T cells and CXCL16-expressing myeloid cells in the choroid plexus and CSF as central elements in this process provides valuable insights into the cellular interactions that may underpin BBB disruption and CNS inflammation in ICANS.

Moreover, our spatial transcriptomics and immunohistochemistry analysis suggested that CXCL16 is primarily produced by myeloid cells within the CNS, proposing a route through which these cells could recruit and stimulate CXCR6 + T cells in regions affected by ICANS. This local increase in pro-inflammatory myeloid cytokines, such as IL-18 [40, 44], which can co-stimulate INF-γ production, further implies a role for these cells in amplifying cytotoxic immune responses, potentially exacerbating neuroinflammation. These insights underscore the possibility of targeting the CXCL16/CXCR6 signaling pathway as a means to attenuate ICANS while preserving the systemic immune responses critical for CAR T cell therapy’s effectiveness. Antibodies and small molecules specifically targeting CXCL16/CXCR6 have previously been developed [45, 46], but have not been tested in clinical trials based on established databases (ClinicalTrials.gov).

In addition, the relative ease of determining CXCR6/CXCL16 at the protein level suggests clinical relevance of this axis as a disease-specific biomarker. However, while it also correlates with disease activity, the impact of different clinical ICANS phenotypes in relation to the CXCL16/CXCR6 axis is still unknown.

A significant limitation of our study is our inability to compare our findings directly with CSF samples from CAR T cell–treated patients who did not develop ICANS due to ethical constraints. As a result, the specificity of CXCL16/CXCR6 axis activation to ICANS remains to be systematically validated in future studies. Additionally, technical variability across independent datasets may influence gene expression profiles and should be considered when interpreting our results. While cryopreservation of CSF cells has been shown to preserve all major cell types, minor changes cannot be ruled out, especially in more sensitive populations such as monocytes and granulocytes [26, 27]. Of note, our cohorts differed significantly in age and sex, which we tried to mitigate by their addition as covariates within our transcriptomic analysis. However, its effect and the role of different ICANS phenotypes is still unknown. It is important to note that our longitudinal study—tracking individual patient markers from pre-CAR T infusion to ICANS onset and remission—was confined to just one patient. This significant constraint underlines the need for cautious interpretation of the temporal dynamics observed in our study.

Moving forward, it remains to be fully determined whether CXCR6 expression directly drives cytotoxic activity or simply marks immune cells predisposed to CNS infiltration. Additionally, the specific molecular mechanisms through which CXCL16+ myeloid cells recruit and activate CXCR6+ CAR T cells require further exploration.

## Conclusions

In conclusion, our study identifies the CXCL16/CXCR6 axis as a contributory factor in ICANS pathophysiology, pointing to the recruitment and activation of cytotoxic CXCR6+CD4 CAR T cells in the CNS as a potential pathway driving neuroinflammation. This insight provides a foundation for potentially using this axis as a biomarker for disease severity and a therapeutic target. While ICANS is currently managed primarily through corticosteroids and IL-6 blockade, our results suggest exploring more targeted interventions aimed at disrupting the CXCL16/CXCR6 axis to possibly prevent or alleviate ICANS more effectively, without impacting the therapeutic benefits of CAR T cell therapy. Further studies are essential to validate the therapeutic potential of targeting this axis and to refine our understanding of its role in the development of ICANS.

## Supplementary Information

Additional file 1: Supplementary figures.

Additional file 2: Supplementary tables.

Additional file 3: Source data of figures.

## Data Availability

Source data are provided with this paper (Additional file 3). All single-cell sequencing data including metadata regarding cluster, celltype, and diseases as well as the Visium HD data have been deposited in the Gene Expression Omnibus (GEO; URL: https://www.ncbi.nlm.nih.gov/geo/query/acc.cgi?&acc=GSE269379) [47].
