# Supplementary material for: The CXCL16/CXCR6 axis is linked to immune effector cell-associated neurotoxicity in chimeric antigen receptor (CAR) T cell therapy
Source: Genome Med. 2025 Jun 30;17:71. doi: 10.1186/s13073-025-01498-6 (PMC12210444; doi:10.1186/s13073-025-01498-6)
Supplement: Supplementary file 1 — Additional file 1: Supplementary figures. [file 13073_2025_1498_MOESM1_ESM.docx]

**Fig. S1**

**
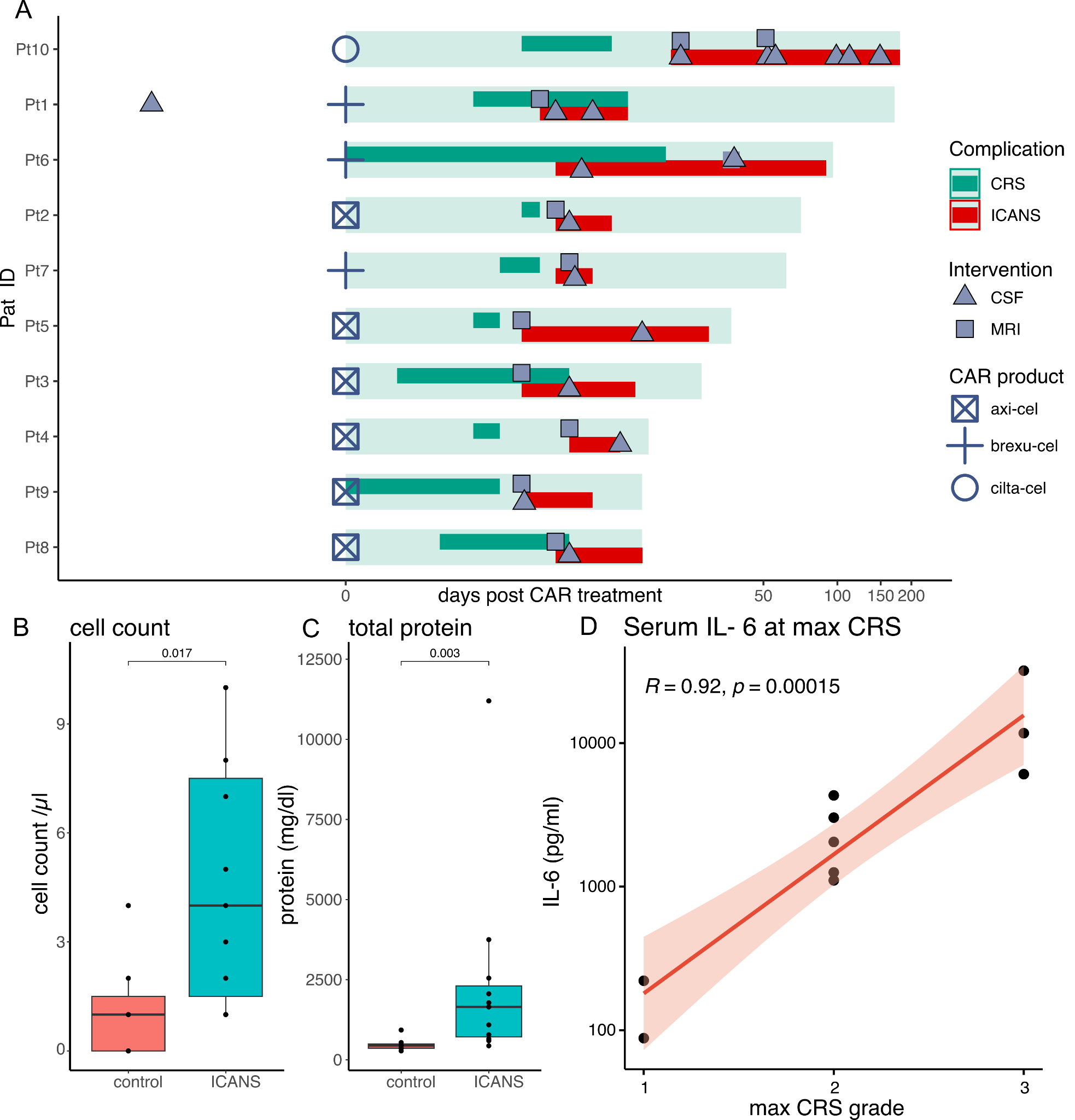
**

**Fig. S1. Disease course of ICANS patients (cohort 1-3)**  **(A)** Swimmer plot depicting the CAR treatment (*brexu-cel* (blue cross), *axi-cel* (blue square), *cilta-cel* (blue circle)) and post CAR treatment follow up (light green bar). Bars depict the clinical signs and symptoms of ICANS (red) and CRS (green). Timeline of CSF withdrawals (CSF) and magnetic resonance imaging (MRI) across the clinical follow up are additionally shown in light blue. Box plots showing **(B)** cell count and (**C)** total protein in the CSF of ICANS and control patients **(D)** Scatter plot showing a correlation (Spearman) of Serum IL-6 levels relative to the max CRS level.

**Fig. S2**

**
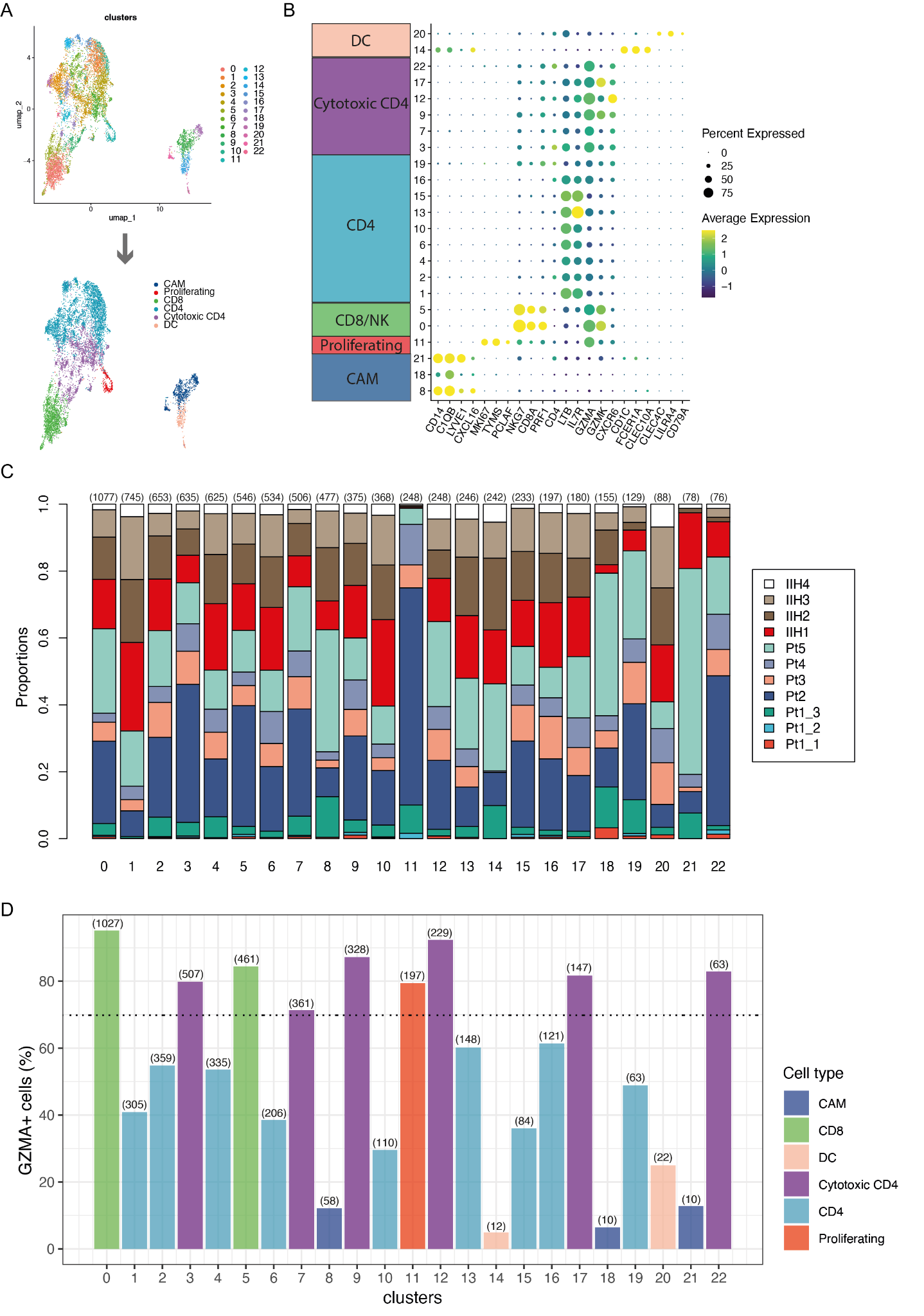
**

**Fig. S2. Classification of CSF cell types.** **(A)** The UMAP plot on the left illustrates 23 color-coded clusters of CSF cells from ICANS patients (N = 7, cell count = 5,148) and IIH control donors (N = 4, cell count = 3,513). These clusters are utilized for the subsequent assignment of cell types depicted in the UMAP plot below. **(B)** Dotplot showing marker genes of cell types. Color encodes average gene expression; dot size represents percentage of cells expressing the gene. **(C)** Cell proportions in clusters across 11 individual samples. Absolute cell counts for each cluster are indicated in parentheses. **(D)** Fraction of GZMA- expressing cells per cell type stratified by clusters. Absolute counts of GZMA+ cells for each cluster are indicated in parentheses.

**Fig. S3**

**
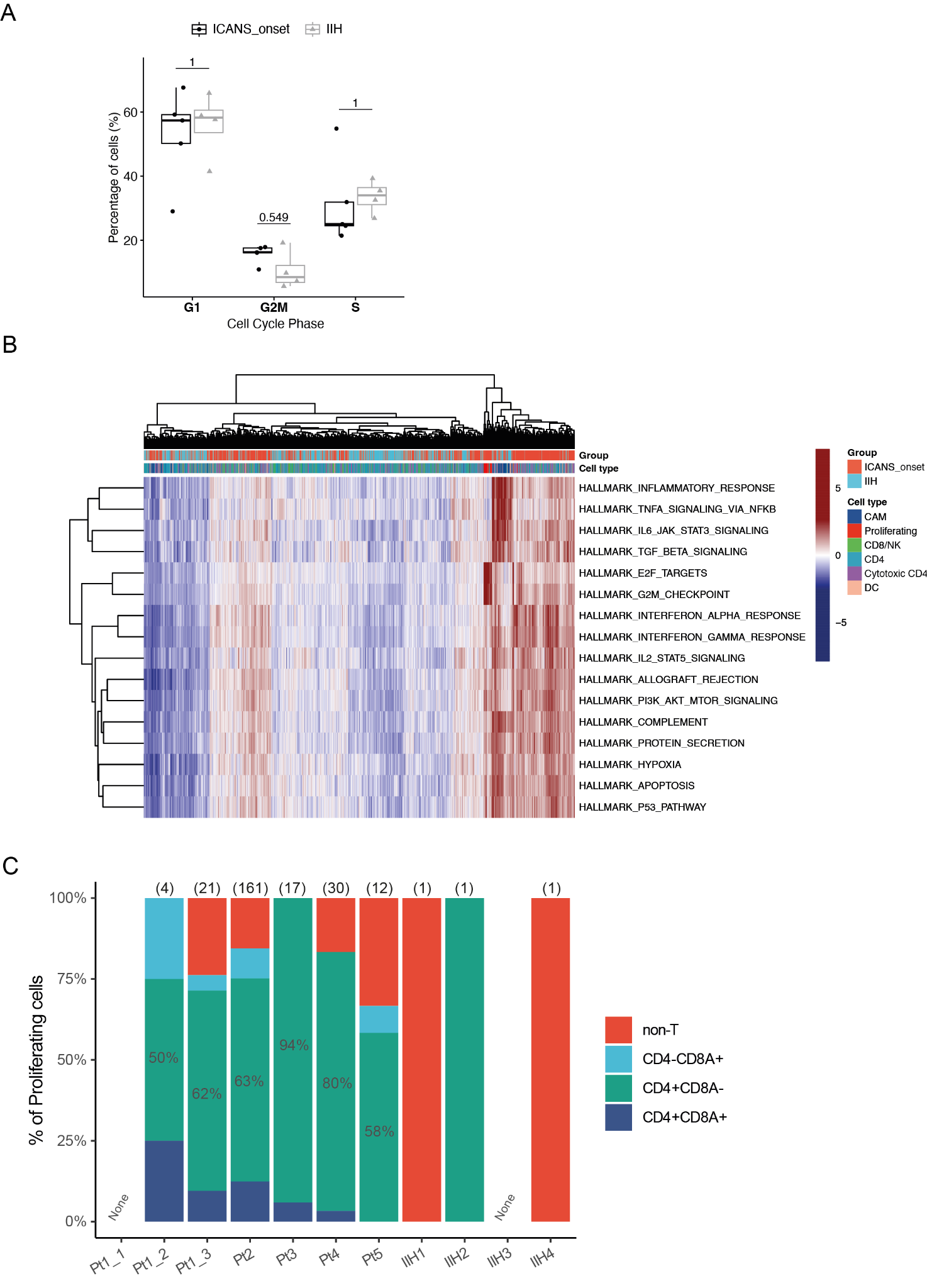
**

**Fig. S3. CSF cell characteristics during ICANS onset.** **(A)** Fraction of cells per sample stratified by cell cycle phases. Each circle/triangle represents a sample. Data are depicted as median, and the lower quatile and upper quartile. Whisker include 1.5 times the interquartile range. Unpaired two samples t-test and the bonferroni method is used to calculate the p-values. **(B)** Heatmap showing gene set enrichment results as estimating ssGSEA scores across all cell types of ICANS_onset patients and IIH control donors. **(C)** Composition of Proliferating cell clusters across samples.

**Fig. S4**

**
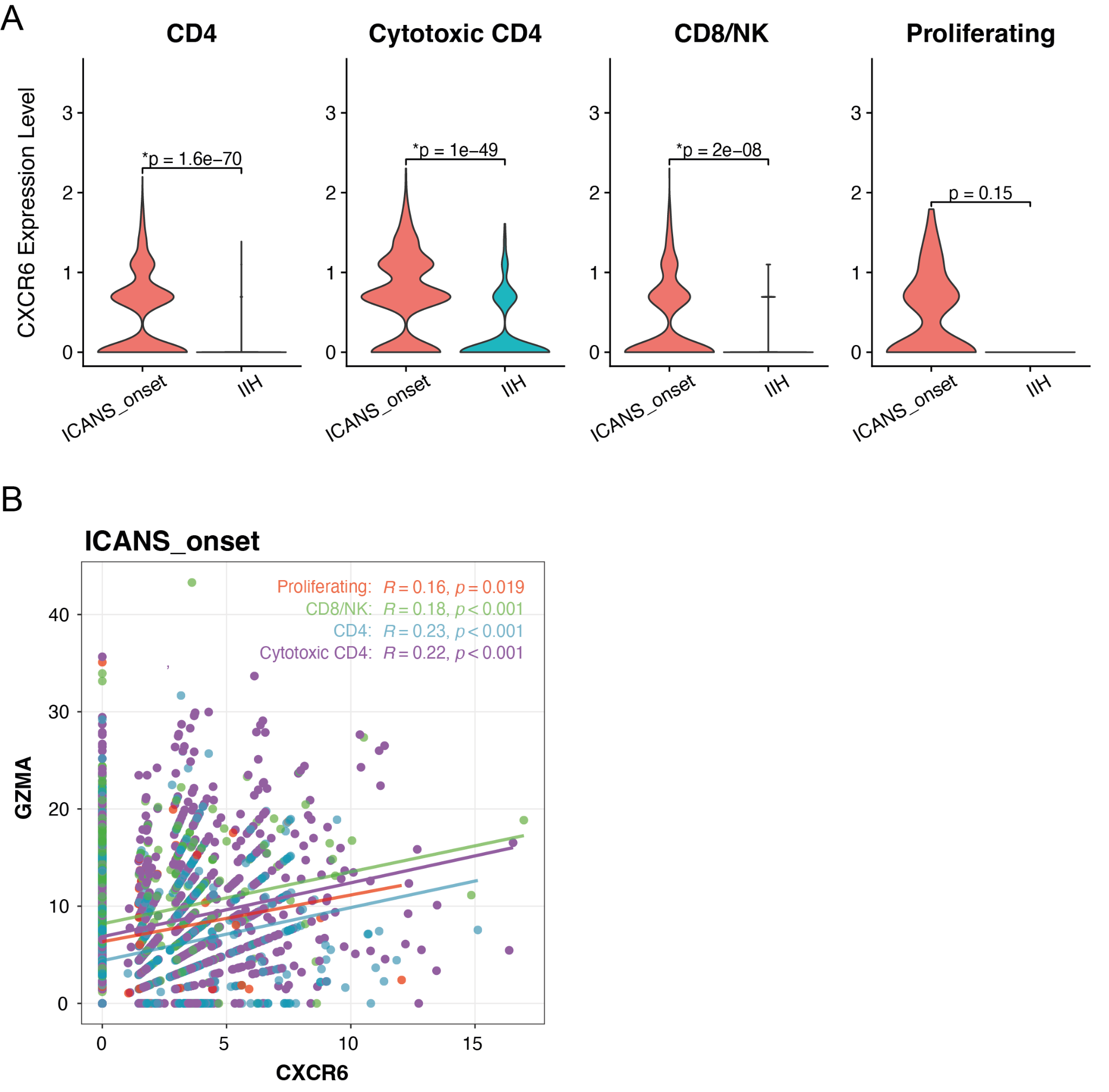
**

**Fig. S4. CXCR6 expression correlates with enhanced T cell cytotoxicity.**  **(A)** Violin plots comparing the normalized CXCR6 expression levels across various T cell subsets between ICANS_onset and IIH samples. **(B)** Correlation plot showing the relationship between CXCR6 and GZMA expression in different T cell populations (grouped and colored by CD4, Cytotoxic CD4, CD8/NK, and Proliferating T cells) from ICANS_onset patients. A significant positive correlation is observed between CXCR6 and GZMA across all T cell populations, with the highest correlation found in CD4 and Cytotoxic CD4 cells (R and p-values indicated). Values on the x- and y-axes represent the percentage of gene counts attributed to CXCR6 (x-axis) and GZMA (y-axis) for each cell. These percentages were calculated using the 'PercentageFeatureSet' function in the Seurat package.

**Fig. S5**

**
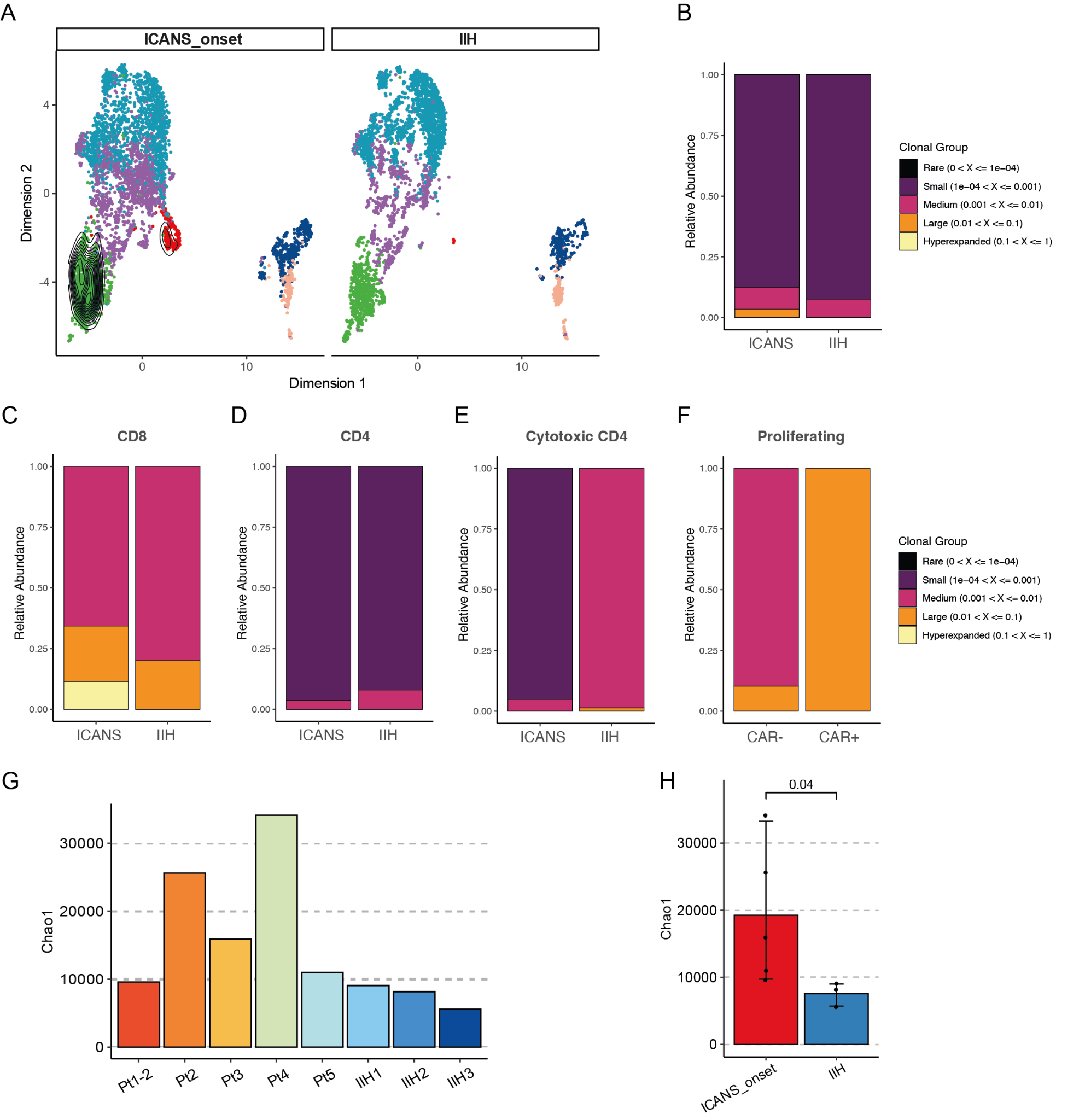
**

**Fig. S5. TCR clonal expression in the CSF T cells during ICANS onset.** **(A)** UMAP plots depicting the distribution of total CSF TCR clones with a clonal frequency over 1%. **(B)** Relative proportion of TCR clones between ICANS_onset and IIH. **(C)** Relative proportion of TCR clones of CD8 cells between ICANS_onset and IIH. **(D)** Relative proportion of TCR clones of CD4 cells between ICANS_onset and IIH. **(E)** Relative proportion of TCR clones of Cytotoxic CD4 cells between ICANS_onset and IIH. **(F)** Relative proportion of TCR clones of Proliferating cells between CAR+ and CAR-. **(G-H)** Diversity analysis compares the diversity of clonotypes in sample groups using the Chao1 index (ICANS_onset: N=5; IIH: N=3). Individual data points are shown using overlaid dot plots. Statistical significance calculated via the Kruskal-Wallis test, as well as the Holm-Bonferroni correction method for p-value adjustments. Mean + SD are shown.

**Fig. S6**

**
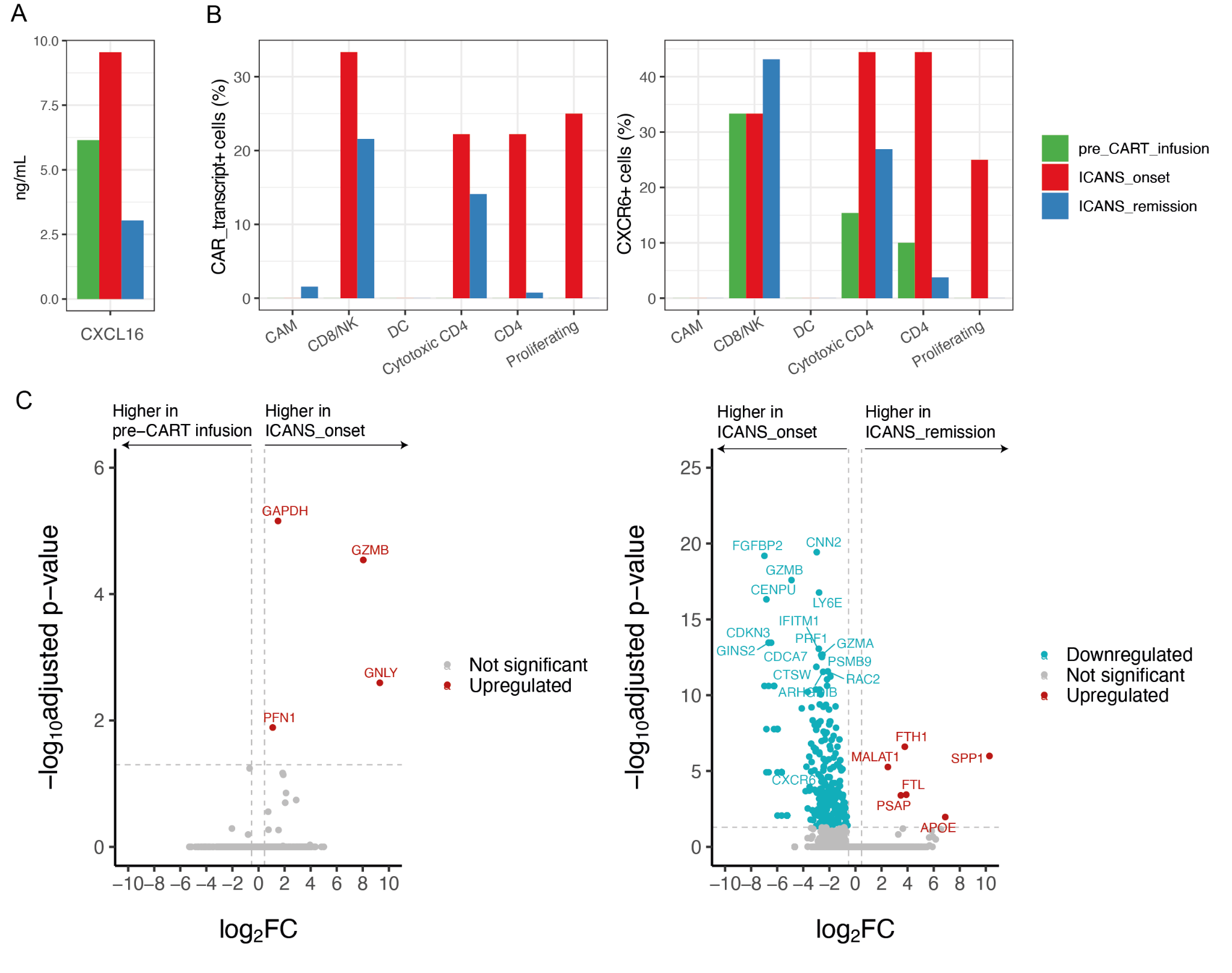
**

**Fig. S6. Longitudinal alteration in the CSF immune landscape.** **(A)** CXCL16 cytokine concentration (pg/mL) measured in the CSF derived from patient ICANS1 during pre-CART infusion, ICANS_onset, and ICANS_remission (N = 1, technical replicates = 2). **(B)** Fractions of CAR_transcript- and CXCR6-expressing CSF cells during pre-CART infusion, ICANS_onset, and ICANS_remission of ICANS1 patient (N = 1) stratified by cell types. **(C)** Differentially expressed genes in ICANS_onset versus pre-CART infusion and in ICANS_remission versus ICANS_onset. Statistical significance was calculated in limma using a moderated t-statistic based on the robust empirical Bayes method in limma. P values were adjusted with the Benjamini-Hochberg method.

**Fig. S7
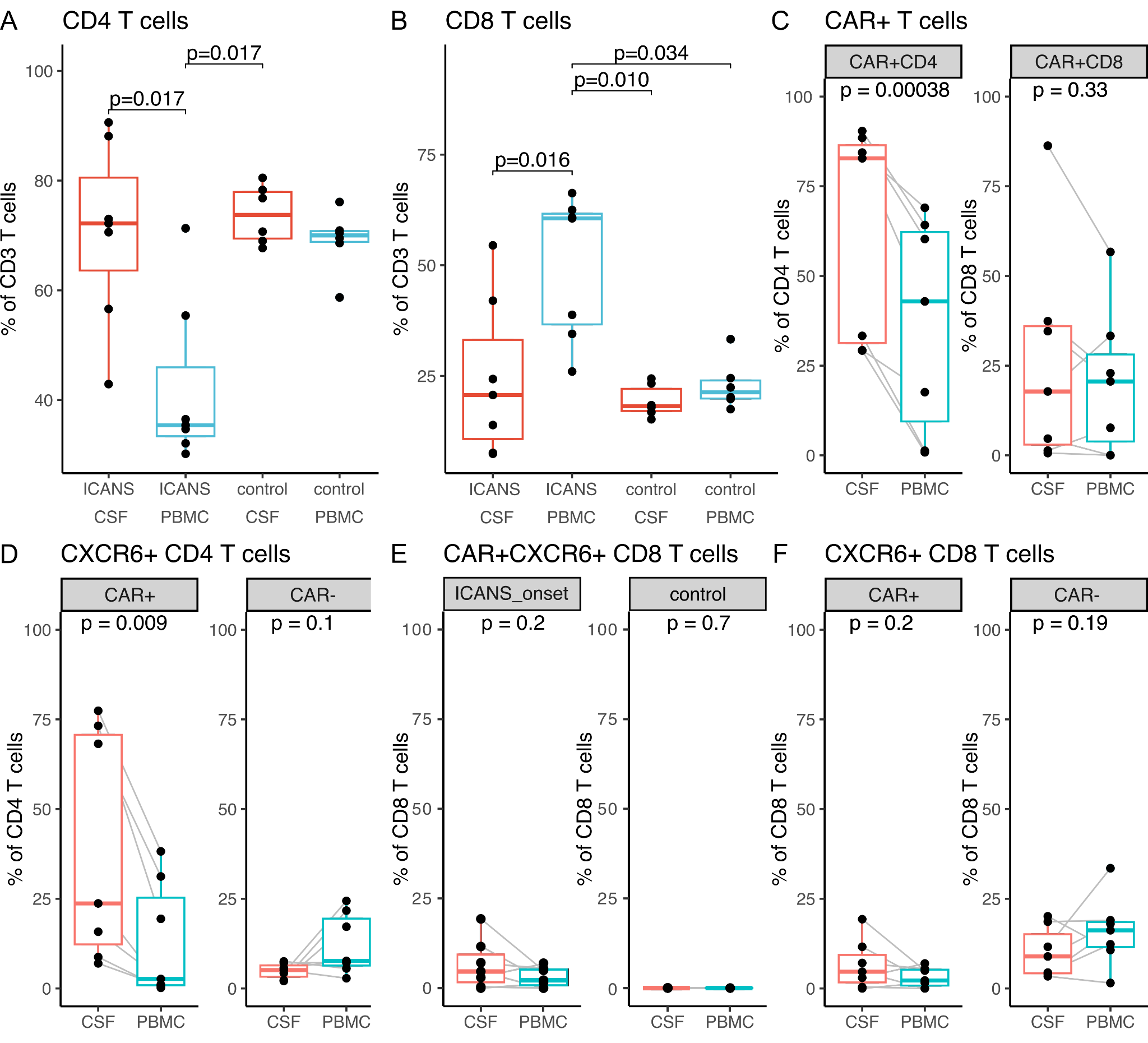
**

**Fig. S7. CXCR6 localizes predominantly on CD4 CAR CSF cells. (A)** CD4 and **(B)** CD8 T cells in the ICANS_onset (N = 7) and control donors (N = 7). **(C)** shows percentage of CAR T cells in CD4 and CD8 populations. **(D)** Comparison of CXCR6+CAR+ and CXCR6+CAR- cells in CD4 T cells. **(E)** Comparison of CAR+CXCR6+CD8 T cells in CSF and PBMC of ICANS_onset and control patients **(F)** Depiction of CXCR6+CAR+ and CXCR6+CAR- cells in CD8 T cells in the ICANS_onset cohort. Boxplots showing percentage of respective population depicted on the y-axis in CSF (red) and PBMC (blue) as measured by flow cytometry. Gating scheme as shown in Supplementary Figure 7. Individual data points are shown using overlaid dot plots, gray lines connect paired samples. Data are depicted as median, and the lower quatile and upper quartile. Whisker include 1.5 times the interquartile range. If more than two groups were compared a Kruskal-Wallis with post hoc Dunn test was performed, if else paired t-test was calculated for statistical significance.

**Fig. S8**

**
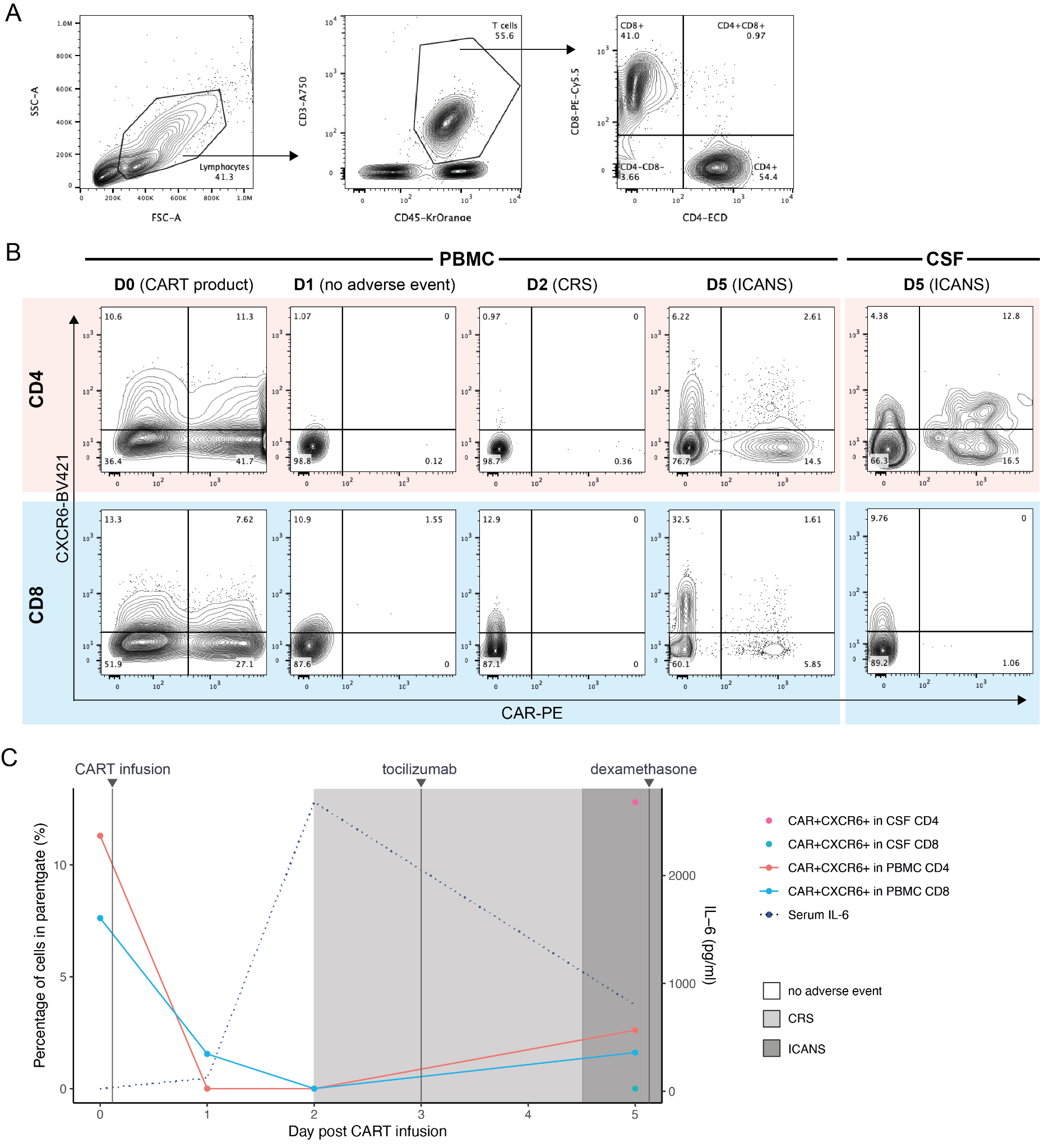
**

**Fig. S8. Longitudinal flow cytometry analysis demonstrates a selective increase in CXCR6+CAR+ CD4 T cells during ICANS but not during CRS. (A)** Gating strategy used to identify CD4 and CD8 T cells. **(B)** Contour plots displaying the expression levels of CXCR6 and CAR in CD4 (top) and CD8 (bottom) T cells on day 0 (D0, CART product), day 1 (D1, no adverse event), day 2 (D2, CRS), and day 5 (D5, ICANS) post-CART infusion, analyzed in PBMC or CSF. **(C)** Line graph illustrating the temporal dynamics of CAR+CXCR6+ CD4 and CD8 T cells, alongside serum IL-6 levels, in relation to days post-CAR infusion. Shaded regions indicate the occurrence of CRS (light grey) and ICANS (dark grey). Vertical lines mark the timing of CAR infusion and the treatments administered during CRS and ICANS.

**Fig. S9**

**
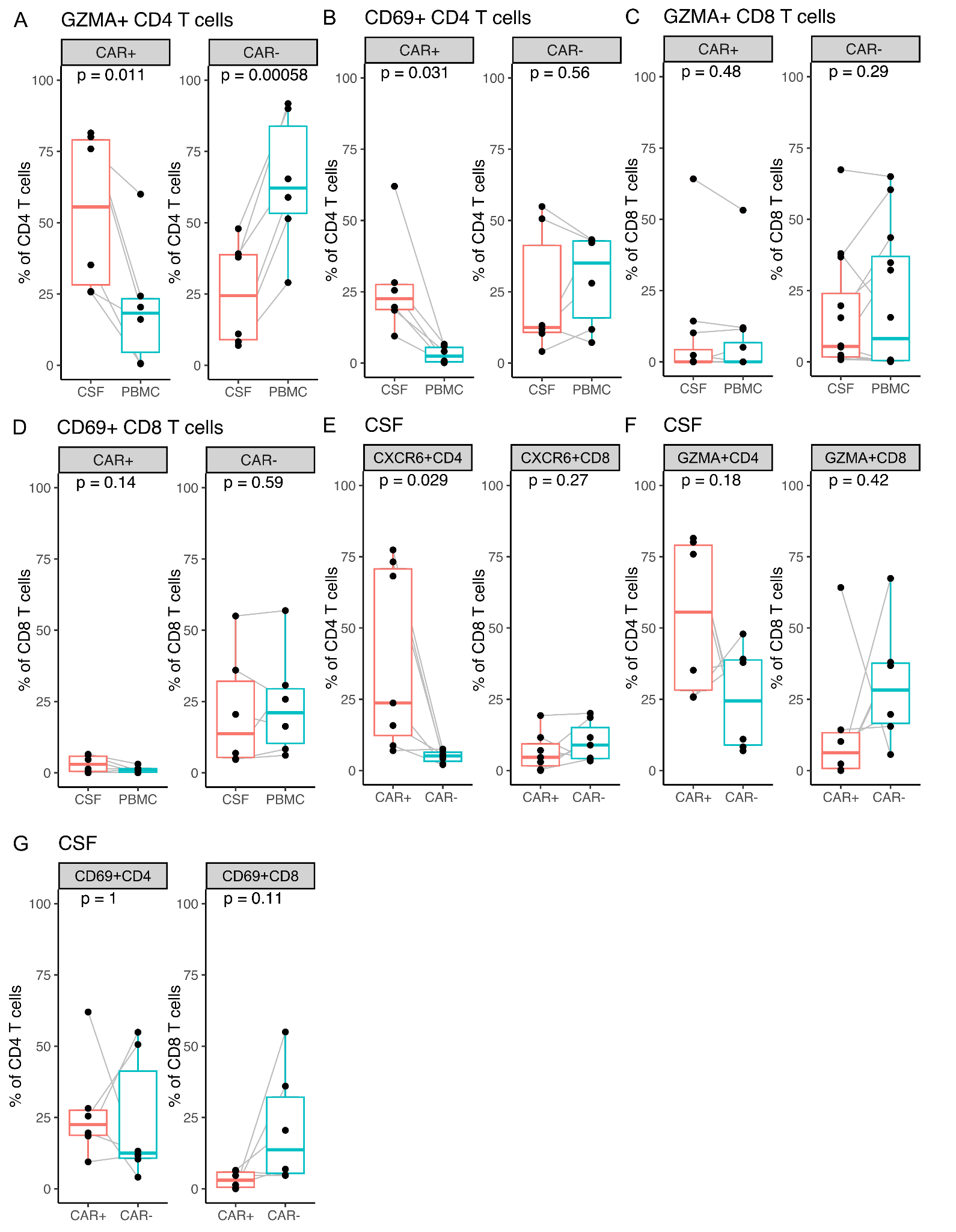
**

**Fig. S9. CSF CD4 CAR cells show a significant increase of activation markers in ICANS_onset patients.** Boxplots showing percentage of **(A, C)** GZMA+ and **(B, D)** CD69+ by CAR status in **(A, B)** CD4 and **(C, D)** CD8 T cells in the ICANS_onset (N = 7) cohort in CSF (red) and PBMC (blue) measured by flow cytometry. **(E-G)** Expression of (E) CXCR6, (F) GZMA and (G) CD69 is compared in CAR positive and CAR negative cells in the CD4 and CD8 T cell compartment of ICANS_onset CSF (N = 7). Gating scheme as shown in Supplementary Figure 8. Individual data points are shown using overlaid dot plots, gray lines connect paired samples. Data are depicted as median, and the lower quatile and upper quartile. Whisker include 1.5 times the interquartile range. A paired t-test was calculated for statistical significance.

**Fig. S10**


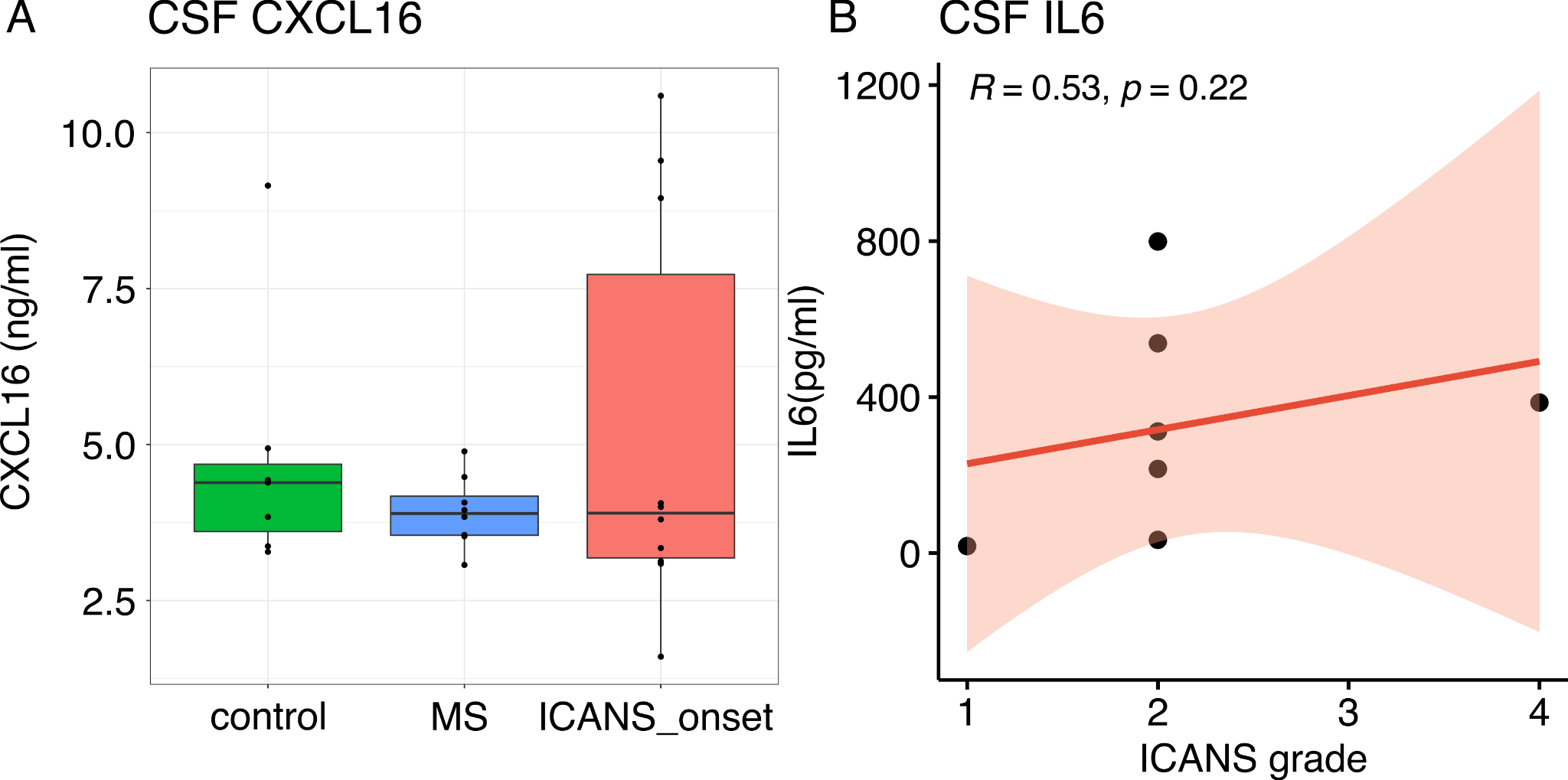


**Fig. S10. IL16 correlates with ICANS severity. (A)** Boxplot representing CXCL16 concentration in ng/ml in the CSF of different cohorts, including control: N = 7, multiple sclerosis (MS): N = 7, and ICANS_onset samples: N = 9. Data are depicted as median, and the lower quartile and upper quartile. Whisker include 1.5 times the interquartile range. **(B)** Scatter plots depicting spearman correlations of CSF IL-6 levels, if available (N = 7) with ICANS grade. The line depicts the linear regression and R is showing the correlation coefficient.

**Fig. S11**

**
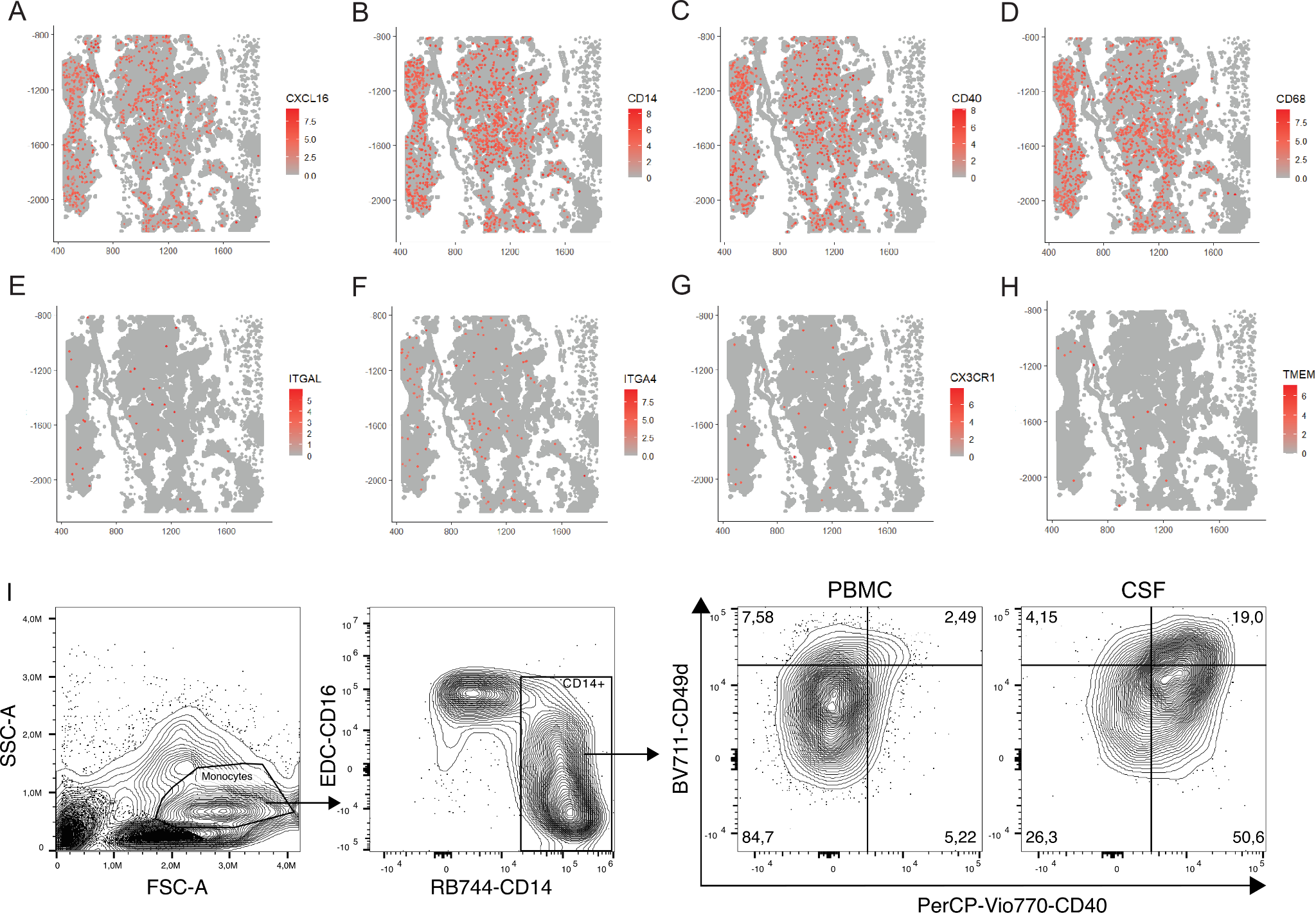
**

**Fig. S11. Phenotyping of myeloid cells in ICANS patients. (A-H)** Spatial transcriptomic of postmortem choroid plexus from an ICANS patient with spatial distributions of **(A)** CXCL16, **(B)** CD14, **(C)** CD40, **(D)** CD68, **(E)** ITGAL, **(F)** ITGA4, **(G)** CX3CR1, and **(H)** TMEM1119 genes in a 6.5 x 6.5 mm capture area with 8-µm pixel size by VisiumHD. **(I)** Explorative flow cytometric analysis with contour plots and gating scheme displaying the expression levels of CD40 and CD49d in monocytes in an ICANS patient, analyzed in PBMC or CSF.

**Fig. S12**

**
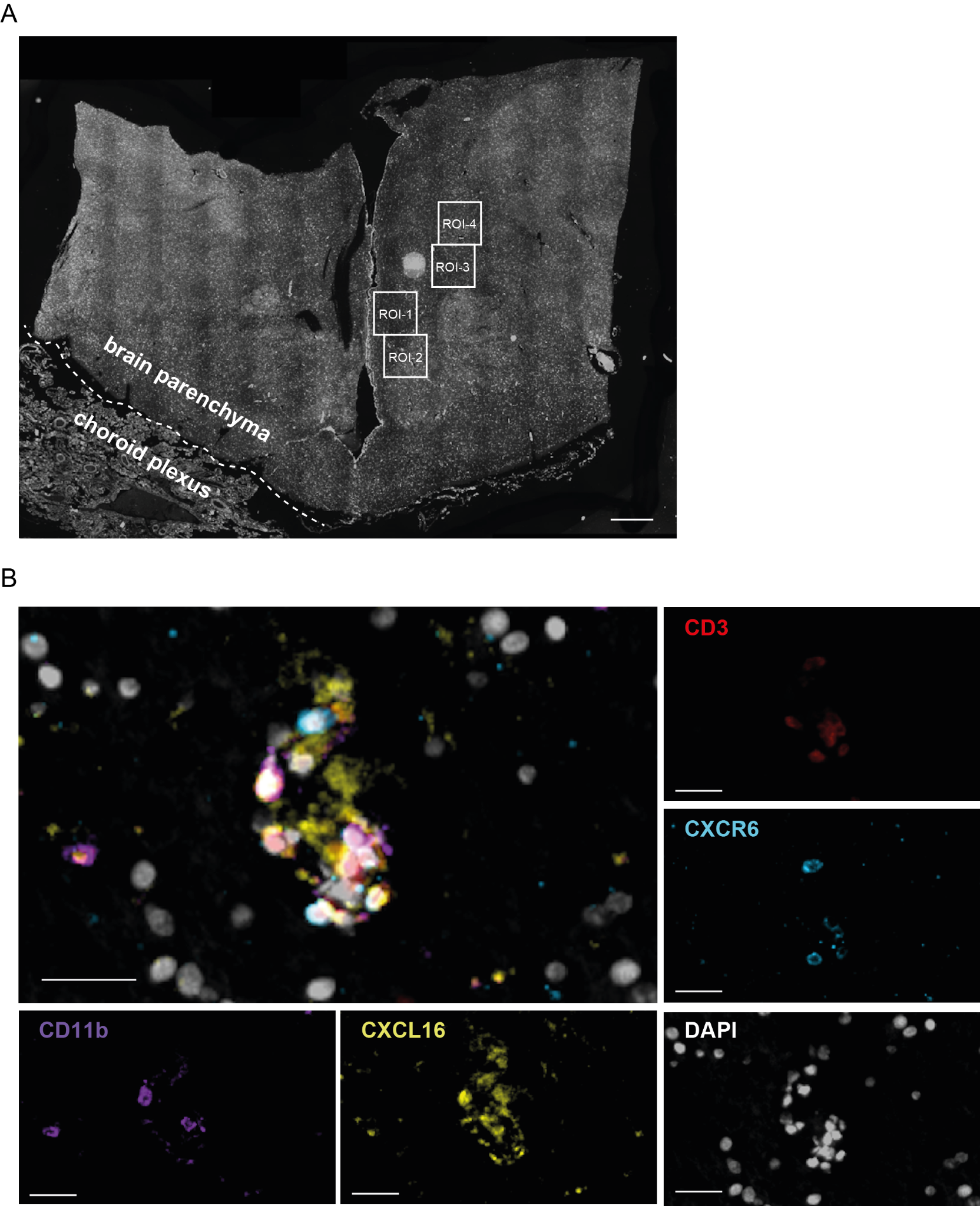
**

**Fig. S12. Detection of CXCL16-CXCR6 axis in the postmortem ICANS brain.** **(A)** Overview image showing four regions of interest (ROIs), each 1000 x 1000 μm, with T cell infiltration. These ROIs were selected for proximity analysis in Fig. 3E and are located within the brain parenchyma of a post-mortem ICANS case (N = 1). Scale bar: 1000 μm. **(B)** Multiplexed immunofluorescence staining in an additional representative field of post-mortem ICANS brain parenchyma. CD3 (red), CXCR6 (light blue), DAPI (white), CXCL16 (yellow), and CD11b (violet). Scale bar: 20 μm.

**Fig. S13**


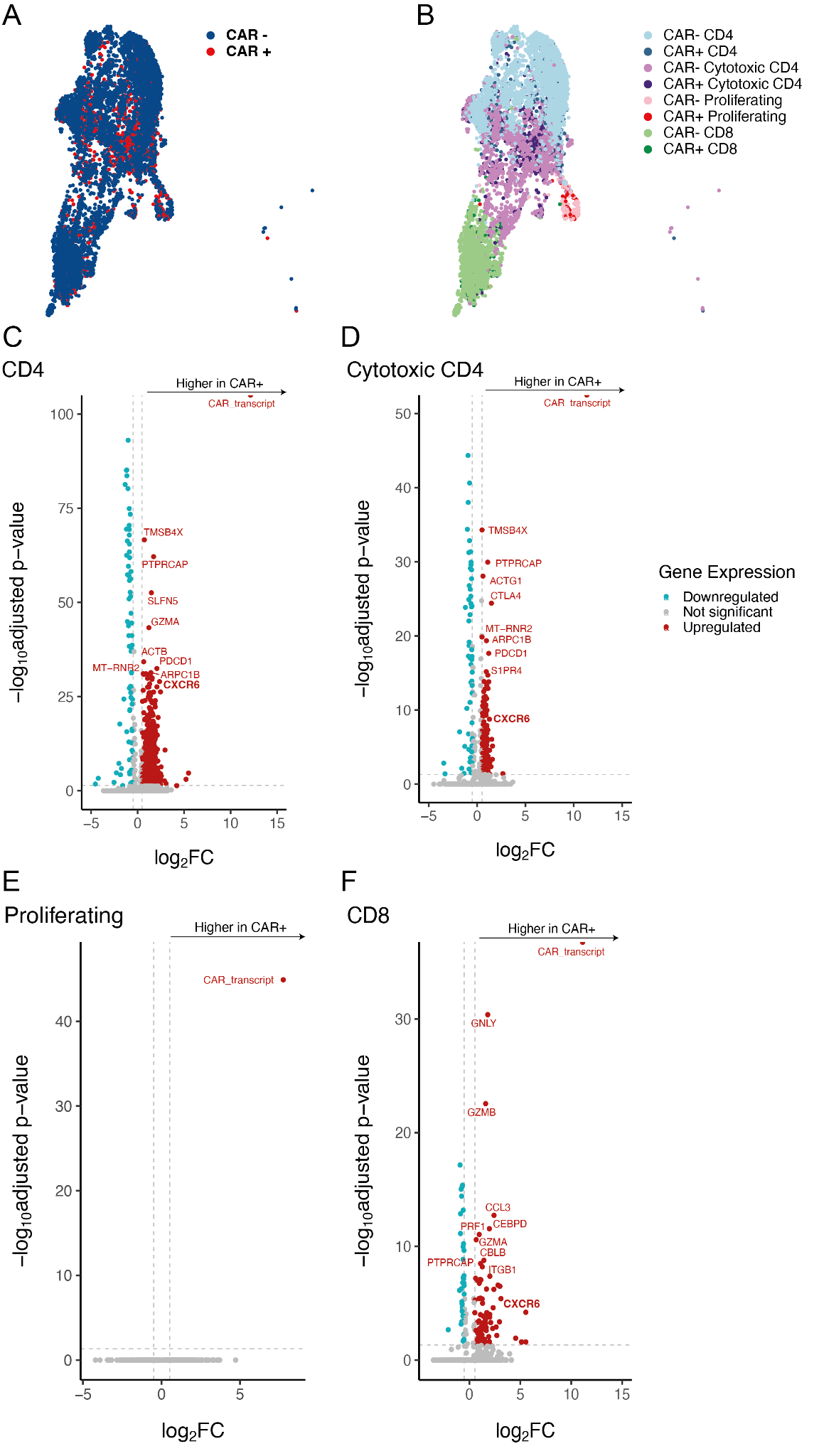


**Fig. S13. Up-regulated genes related to CAR-transcript expression in the CSF T cell subsets in ICANS_onset patients.** **(A-B)** UMAP representation of dataset from ICANS_onset patients (N = 5, cell count = 4,716) and IIH control donors (N = 4, cell count = 3,513), colored by CAR-transcript expression (A) and CAR+ or CAR- T cell populations (B). **(C-F)** DEGs with top markers and CXCR6 highlighted in all T cell populations (CD4, Cytotoxic CD4, Proliferating, and CD8) of CAR+ versus CAR- cells. The horizontal lines represent the significance thresholds (horizontal dashed line: *P* = 0.05).
